# The Mechanism of Action of Biguanides: New Answers to a Complex Question

**DOI:** 10.3390/cancers14133220

**Published:** 2022-06-30

**Authors:** Laura Di Magno, Fiorella Di Pastena, Rosa Bordone, Sonia Coni, Gianluca Canettieri

**Affiliations:** 1 Department of Molecular Medicine, Sapienza University of Rome, 00189 Rome, Italy; laura.dimagno@uniroma1.it (L.D.M.); fiorella.dipastena@uniroma1.it (F.D.P.); rosa.bordone@uniroma1.it (R.B.); sonia.coni@uniroma1.it (S.C.); 2 Istituto Pasteur—Fondazione Cenci—Bolognetti, 00161 Rome, Italy

**Keywords:** biguanides, complex I, metabolism, redox, metformin, cancer

## Abstract

**Simple Summary:**

In the last two decades, the antidiabetic drugs, biguanides, have received considerable interest owing to their presumed antitumor properties. A critical issue that has been at the center of many studies is how they act at the molecular level. Most works propose that biguanides inhibit mitochondrial complex I, which causes ATP depletion and activation of compensatory responses, responsible for the therapeutic properties. However, complex I can only be inhibited with concentrations of biguanides that cannot be tolerated by animals and patients, suggesting that alternative targets and intracellular perturbations are involved. Here, we will discuss the current knowledge of the mechanisms of action of biguanides, when used under clinically relevant conditions. The ongoing clinical trials in cancer and the proper conditions of usage will also be addressed. Understanding the mode of action of these drugs represents critical information for further investigation and usage in cancer models.

**Abstract:**

Biguanides are a family of antidiabetic drugs with documented anticancer properties in preclinical and clinical settings. Despite intensive investigation, how they exert their therapeutic effects is still debated. Many studies support the hypothesis that biguanides inhibit mitochondrial complex I, inducing energy stress and activating compensatory responses mediated by energy sensors. However, a major concern related to this “complex” model is that the therapeutic concentrations of biguanides found in the blood and tissues are much lower than the doses required to inhibit complex I, suggesting the involvement of additional mechanisms. This comprehensive review illustrates the current knowledge of pharmacokinetics, receptors, sensors, intracellular alterations, and the mechanism of action of biguanides in diabetes and cancer. The conditions of usage and variables affecting the response to these drugs, the effect on the immune system and microbiota, as well as the results from the most relevant clinical trials in cancer are also discussed.

## 1. Introduction

The history of biguanides began in the 19th century when it was found that the blood-glucose-lowering properties of the herb *Galega officinalis* (French lilac), used since the medieval age to treat polyuria and other diseases, were due to galegine, a derivative of guanidine contained in the plant seeds and flowers. The identification of galegine led to the synthesis of various biguanides (synthelin A and B, biguanide, metformin, phenformin, and buformin) in the early 20th century that were tested as antidiabetic agents but shortly discontinued due to toxicity issues or presumed low potency.

Starting from the 1980s, further studies led to the re-evaluation of the use of metformin in type 2 diabetes mellitus (T2DM), providing strong evidence for its effectiveness and safety [1] and leading to FDA approval in 1994. Since then, metformin has progressively gained ground, to become the most widely prescribed oral antidiabetic drug and first-line therapy for the treatment of T2D in the last two decades.

The broad utilization of metformin has also allowed epidemiological observations reporting a significant reduction of the risk of cancer in diabetic patients treated with this drug [2,3,4], which has prompted a significant effort aimed at establishing the therapeutic efficacy of metformin against cancer in cells culture, animal models, and patients.

Phenformin and buformin were prescribed for the treatment of T2D starting from the 1950s but were withdrawn from the market in the 1970s because of the higher risk of cardiac mortality and lactic acidosis [5,6]. Following the increased interest in the anticancer properties of metformin, these drugs (particularly phenformin) have been re-considered for cancer treatment, showing significant antitumor effects, often stronger than metformin, in numerous preclinical studies, likely related to their higher cell permeability.

A substantial amount of effort has been devoted to understanding how biguanides act at the molecular level, an issue that has not yielded unique conclusions but rather has been quite controversial.

According to a widely accepted interpretation, the primary target of biguanides underlying both their antidiabetic and anticancer effects, is mitochondrial complex I of the respiratory transport chain. All biguanides display an inhibitory effect on complex I and inhibit the rate of oxygen consumption, thereby causing energy stress, increase in AMP/ATP ratio, and activation of AMP Kinase (AMPK), which is believed to be a master mediator of the therapeutic effects of these drugs, through phosphorylation-mediated regulation of key targets such as hepatic CRTCs in diabetes and mTOR in tumors.

However, while this model is generally accepted and used to support many experimental findings, it has also raised concerns that have led to alternative interpretations.

A primary reason for this lack of consensus is the inconsistencies in the drug concentrations and conditions used in the experimental settings.

Indeed, most of the reported mechanisms of action of biguanides have been demonstrated in cell culture using doses of the drugs and culturing conditions that are different from those found in patients or animal tissues. Even if many studies have shown that these parameters have a profound influence on the drug response, these pieces of information have been often poorly considered when addressing the mechanism of action of these drugs.

In the first part of this article, we review the available information about the pharmacological properties of biguanides, describing the structure, the dosage administered in patients and animal models, the concentrations reached in the circulation and tissues over time, the cellular transporters, and how these drugs travel across the cells. In the second section, we illustrate what is known about the mechanism of action of these drugs as glucose-lowering agents, in terms of target tissues and target molecules. In the third part of this work, we describe the current knowledge on the mechanism of action of biguanides in cancer, the variables affecting the cellular responses, and the available data arising from clinical trials.

## 2. Pharmacological Properties

Biguanides are a class of compounds in which two guanidine groups are bound by a common nitrogen atom. They all share the feature of being both polar and hydrophilic molecules, highly soluble in aqueous media because of two imino and three amino groups in tautomerism. However, they also differ in some chemical peculiarities, responsible for the pharmacokinetic and pharmacodynamic properties in each of them.

### 2.1. Metformin: Uptake, Therapeutic Concentration, Excretion

Metformin (3-(diaminomethylidene)-1,1-dimethylguanidine) carries two methyl substituents in position 1 and is synthesized from 2-cyanoguanidine and dimethylammoniumchloride [7]. The first evidence of the hypoglycemic activity of metformin in animal models was from Slotta and Tschesche in 1929 [8], and its clinical use was first reported by Sterne in 1957 [9]. In type 2 diabetic patients, metformin is administered orally as immediate or extended-release tablets. The immediate release is generally taken 2–3 times a day, while the extended release is administered once daily.

The daily dose ranges from 500 to 2550 mg. Metformin is rapidly dissolved in the gastrointestinal tract [10] but, due to its hydrophilic nature, the absorption cannot occur passively through the plasma membrane but requires active transport. Multiple organic cation transporters are involved in the uptake of metformin, and many of them are important in its pharmacological action, as mediators of metformin entry into target tissues. Metformin is a substrate of various organic cation transporters (OCT), including OCT1 (SLC22A1), OCT2 (SLC22A2), OCT3 (SLC22A3), MATE1 (SLC47A1), MATE2 (SLC47A2), PMAT (SLC29A4), and OCTN1 (SLC22A4) [11,12,13,14,15,16]. Several transporters have been implicated in metformin intestinal absorption. PMAT is primarily located on the apical membrane of polarized epithelial cells [17], while transporters in the SLC22 family are expressed in the small intestine and play a role in metformin absorption.

Metformin can also cross the enterocytes through the organic cation transporters 1 and 3 (OCT1 and OCT3) [18,19]. OCT3 is localized in the apical membrane and carries metformin into enterocytes, while OCT1 is localized in basolateral membranes and transports metformin into the interstitial fluid [20]. OCT1 and 3 are also expressed on the basolateral membrane of hepatocytes and mediate metformin liver uptake [21]. High expression of OCTs is responsible for the elevated metformin accumulation in mouse liver (~40 μM) when compared to serum (~5 μM) [22]. In agreement with this value, Ma and colleagues recently showed that in mouse primary hepatocytes treated with 5 μM metformin for up to 48 h, the intracellular concentration was 25–40 μM, suggesting the ability to accumulate 5–8-fold in these cells [23]. Similarly, Moonira et al. measured an intracellular/extracellular metformin concentration ratio of about 5-fold after 3 h incubation of mouse hepatocytes with 100–200 μM of the biguanide [24].

OCT transporters could also move metformin from the liver to the blood thus causing its rapid distribution into peripheral body tissues and fluids. Both OCT1 and 3 are also expressed in skeletal muscles where they mediate metformin uptake.

Metformin does not bind to plasma proteins, and this causes its rapid distribution throughout the body [25] (Table 1). The plasma concentration measured in subjects taking 1.5–2.5 g of metformin orally per day (~30 mg/kg/day) ranges between 4 and 15 μM [10]. In particular, 3 h after receiving a single dose of 0.5 g metformin orally, the peak plasma concentration ranges between 7.74–12.39 μM; 3 h after a single dose of 1.5 g metformin, the peak plasma concentration is 23.23 μM. Assumption of 1 g metformin twice a day determines a plasma mean concentration of 3.1–10.07 μM. The mean concentration over a dosage interval is 6.66 μM. Lalau et al., in 2003, measured metformin plasma concentration in subjects with type 2 diabetes mellitus under metformin therapy within the recommended dosage range (1700–2550 mg/day) and reported a mean metformin concentration of 3.8 μM [26]. 

Madiraju et al. in 2018 detected metformin plasma concentration in human subjects 3 h after 1 g of metformin by oral administration, and values ranged from 14 μM to 22 μM [27]. In contrast to the rapid decrease of plasma concentrations, Bailey et al. detected metformin accumulation in the gut after administering 850 mg daily for 2–3 weeks and then twice daily for other 3–5 weeks to T2 diabetes patients [28]. Metformin levels detected 12–16 h after the last 850 mg dose (pre-dose jejunal sample) corresponded to 33 ± 26 ng/mg wet weight of tissue (approximately 250 μmol/kg), while the concentration reached 3 h after the last 850 mg morning dose (post-dose sample) was 504 ± 232 ng/mg wet weight of tissue (approximately 4 mmol/kg). These values were 30–300 times higher than metformin plasma concentration (8–24 μM).

Pentikainen and colleagues [29] measured metformin plasma concentration in three patients, following i.v. injection of 500 mg [29] After 1 h, they observed a peak of 5 μg/mL (=38.68 μM), which rapidly decreased at 1.5 μg/mL (=11.6 μM) 2h after administration. Renal clearance after intravenous administration calculated in this study (454 ± 47 mL/min) was comparable to that calculated after oral administration (507 ± 129 mL/min) by Graham et al. in 2011 [10].

Data on liver concentrations of metformin in humans are not available and it is, therefore, difficult to establish the exact therapeutic values. However, based on a presumed three-fold higher liver concentration compared to plasma content (where the calculated range is 20–30 μM), the estimated hepatic exposure is believed to be 60–90 μM, corresponding to 2 g/day in patients or to oral dosing in rodents of 50–100 mg/kg [30].

As for dosing in rodents, other authors indicate that the oral dose of 250 mg/kg/day in mice corresponds to 30 mg/kg/day in humans (2–2.5g/day), considering the interspecies scaling in pharmacokinetics [22]. 

Metformin concentration reached in plasma and tumor tissue ranges from 3.2 to 12.4 μM [22]. These data are consistent with work from Madiraju and colleagues where after ad libitum administration of 200–300 mg/kg/day of metformin, plasma concentration was 15 μM and liver concentration was 40 μM [27].

Madiraju et al. also reported that 30 minutes after intravenous injection of 50 mg/kg metformin in rats, plasma concentration was 74 μM, while in the liver it was 100 μM.

Chandel and collaborators [22] observed that administration of 350 mg/kg metformin by oral gavage for 3 weeks caused a peak of 1500 μM in the liver and 200 μM in the tumor, in a mouse model of lung adenocarcinoma. However, these concentrations were considered supra-pharmacological by others [30]. Time-averaged plasma concentration was 47 μM. The authors also injected metformin 350 mg/kg intraperitoneally (i.p.) for 2 weeks and detected a concentration peak of 100 μM in the liver and tumor after 25 h from the last administration, while the time-averaged plasma concentration was 7.5 μM [22]. Acute IP administration is reported also by Dowling and colleagues [31] after 30 minutes from an injection of 125 mg/kg metformin, mean plasma concentration was 184 μM and it decreased to 42 μM after 1 h.

Wilcock and Bailey, in 1993, analyzed metformin concentration reached in tissues such as the liver and gut after acute administration (50 mg/kg) via oral gavage or intravenous route [32]. Oral gavage seems to be more efficient in achieving higher concentrations: 51.7 μM measured in hepatic portal vein after 30 minutes vs 21.9 μM in inferior vena cava detected after the intravenous injection. Moreover, liver tissue concentration was 37 μM after oral gavage and 22 μM after i.v. As for the gut, they measured different grades of accumulation along the tract with a maximum of 1206 μM and a minimum of 147 μM after oral gavage; the highest concentration reached in the gut after i.v. injection was 55 μM and the lowest 38 μM.

Metformin is not metabolized and is secreted unmodified by the kidney [33], after being transported through OCT2 [34] located on the basolateral side of renal tubular cells. The multidrug and toxin extrusion (MATE) transporters, such as MATE1 and MATE2K contribute to the transport of metformin into urine [35]. The mean renal clearance rate is around 552–642 mL/min. Its mean plasma elimination half-life is 1.5–4.7 h [25,36].

Metformin renal clearance decreases along with the impairment of kidney function and depends on the genetic pool of transporters expressed in kidney cells: OCT2 is the principal carrier involved in the uptake from tubular cells, and OCT1 mediates its secretion but it could also participate in the entry process. OCT3 is also expressed in the kidney. MATE1 is thought to carry metformin out of tubular cells and into the urine, while MATE2K could be the principal extrusion transporter [35,37]. Metformin is excreted in the urine unchanged, and no metabolites have been reported.

### 2.2. Phenformin: Uptake, Therapeutic Concentration, Excretion

Phenformin (1-(diaminomethylidene)-2-(2-phenylethyl)guanidine) is a phenethyl biguanide derivative of metformin that is characterized by the substitution of one of the terminal nitrogen atoms with a 2-phenylethyl group. Phenformin is obtained by heating phenethylamine and cyanoguanidine (37% yield) [38]. It was used as an antidiabetic agent but was later withdrawn from many countries because it was associated with a greater incidence of lactic acidosis [1,39].

Phenformin is more lipophilic than metformin and therefore it is generally thought to passively cross the cell membrane and display a higher potency. This makes phenformin less dependent on active transport, while metformin requires transporters to enter cells [40]. Supporting this idea, Hawley et al., [41] showed that while OCT1 is required to transport metformin in rat hepatoma cells, it is not necessary for phenformin uptake. Other studies revealed that an active phenformin transport is required to cross the mitochondrial membrane: Shitara et al., in 2013, described the role of organic cation/carnitine transporter 1 (OCTN1) in mitochondrial accumulation of phenformin [42]. Bridges et al. (2016) confirmed these data (selective transport) by comparing the ability of biguanides with the same lipophilicity in mitochondria entry [43]. Moreover, work by Sogame et al., (2013) revealed that phenformin has an affinity for hOCT2, which is majorly involved in biguanides uptake from blood to kidney cells, stronger than metformin [44].

Phenformin is rapidly absorbed after oral administration and is not significantly bound to plasma proteins [45] (Table 1). It undergoes hydroxylation in the liver to 4-hydroxy-phenformin [46]. The half-life of circulating phenformin is about 11 h [47]. Both phenformin and its hydroxylated metabolite are predominantly eliminated in the urine [46].

Beckmann and colleagues measured a phenformin plasma concentration of 0.97 μM 2 h after a single oral dose of 100 mg in patients, with a half-life of 3.2 h [45]. Matin et al. also measured phenformin concentration after administration of 100 mg to a diabetic patient. Maximum plasma concentration, measured with mass spectrometry, was reached after 3 h and was 147 ng/mL, corresponding to 0.72 μM [48]. Nattrass et al. in 1980 [49] measured a sustained release formulation of 50 mg of phenformin administered to six healthy volunteers. The values obtained from their analysis (0.19 μM 3.5 h after ingestion) were lower than those obtained by Beckmann and collaborators, although the authors pointed out that the time course could have been altered using a sustained-release capsule. Similar steady-state concentrations were reported by Marchetti and Navalesi [50] and were between 0.13 and 0.56 μM.

More data about phenformin circulating levels in patients were reported by Karam et al. [51]. They commented on the results obtained from the University Group Diabetes Program (UGDP) in which patients with hyperglycemic reactions to oral glucose assumption were treated with different anti-diabetic drugs. Circulating phenformin concentrations measured in these patients using gas chromatography were comprised between 102–241 ng/mL (0.5–1.17 μM).

As for animal models, in a recent work, HPLC analysis was used to measure plasma phenformin in C57BL/6J mice. After 10 days of treatment with phenformin (300 mg/Kg/day) in the drinking water, a 1.4 μM phenformin concentration was detected in the blood [52]. The same dose of 300 mg/Kg/day in the drinking water was used also by Huang et al. [53] and Appleyard et al. [39] for xenografts experiments.

After i.v. administration in the tail vein of 12.5 mg/kg phenformin, the maximum concentration (3.4 μM) was achieved 30 minutes after injection (maximum tolerated dose), while higher doses were not tolerated [52]. 

Phenformin blood concentrations in mice treated for 5- and 7-days ad libitum per os are described also in Shackelford et al. [54] and are in a range between 1–1.5 μM. Similar data were obtained by Bando et al. in 2010 [55] using oral gavage to administer different phenformin doses. Here, 28 days after daily ingestion of 200 mg/kg phenformin, the mean plasma concentration was 1.49 μM.

Various studies have been carried out in animal models highlighting the higher accumulation of the drug in the liver and gut. Wick et al. in 1960 [56] administered 100 mg/kg of phenformin orally or intraperitoneally. In the first case, they detected the maximum liver concentration (2 mM tissue water) after 1h and the maximum GI concentration (3.1 mM tissue water) after 2 h. The intraperitoneal route determined a liver Cmax of 2.6mM tissue water after 2 h and a GI tract Cmax of 0.9 mM after 1 h.

Sogame et al. in 2011 measured phenformin levels in rats after oral gavage administration of 50 mg/kg. The portal vein and liver concentrations after 30′ from ingestion were 2.5 μM and 147.1 μM respectively, while the highest plasma concentration (3.49 μM) was reached 4 h later [57].

Another study by Conlay (1977) measured phenformin serum concentration in patients manifesting lactic acidosis [58]. They received 50 mg of phenformin three times a day. Five of seven patients presented phenformin concentration under 241 ng/mL (1.17 μM) confirming the previous data.

Phenformin is partially metabolized in the liver in N1-p-hydroxy-β-phenethyl biguanide by CYP2D6, and about one-third is excreted in this form, whereas the other two-thirds are eliminated unmodified. It is reported in Beckmann [45] that the maximal excretion rate is 4.1 mg/h. The average half-life of excretion is 3.2 h, which is equivalent to an average rate constant of 0.22 mg/h.

Although the use of phenformin has been discontinued due to the high incidence of lactic acidosis, many studies demonstrated that the increased frequency may be principally related to the subjects receiving this drug. First, kidney dysfunction, which is often associated with diabetes, may reduce the clearance of the drug. Second, some genetic features, such as the expression level of transporters involved in phenformin excretion (OCT2 or MATE), may also affect its plasma levels. Third, alterations of the enzymes that metabolize phenformin (CYP2D6 and P-glycoprotein) can modulate phenformin circulating levels and consequentially the risk of lactic acidosis. Indeed, it has been demonstrated that patients that are poor CYP2D6 metabolizers show higher levels of phenformin plasma concentrations that lead to higher toxicity [59]. The risk of lactic acidosis is also increased by CYP2D6 gene mutations that lead to high levels of unmetabolized phenformin [1,59].

### 2.3. Buformin: Uptake, Therapeutic Concentration, Excretion

Buformin (2-butyl-1-(diaminomethylidene)guanidine) was synthesized and tested as a hypoglycemic agent in the 1950s [60]. Like phenformin, this drug is more lipophilic and effective than metformin, but the major limitation to its usage is the associated high risk of lactic acidosis [61]. For this reason, buformin was withdrawn from clinical use in the 1970s in most countries (except for Romania where it is still commercially available and administered in doses ranging from 50 mg to 300 mg daily).

Buformin is not metabolized [62,63,64] and only 10% has been found to interact with serum proteins [64,65] (Table 1). Data regarding buformin concentration and pharmacokinetics are reported by Lintz et al. [66]. Four diabetic patients were treated with 50 mg of ^14^C-butylbiguanide intravenously. 1 h and 5 h after administration, buformin plasma concentrations were 1.8–2.13 μM and 0.45–0.64 μM, respectively. The biological half-life of butylbiguanide calculated after intravenous administration was 3.7–6.0 h with a mean of 4.6 h. In this study, the authors also measured the total clearance, which ranged from 439 to 618 mL/min, and averaged 536 mL/min. A mean value of 72.4% (61.2–90.2%) of the administered drug was excreted in the urine. Mean renal clearance was 393 mL/min (282–518 mL/min). The value given here for the total clearance (536 + 78 mL/min) corresponds to previously described values. Buformin renal clearance was significantly higher than insulin clearance, and this suggested that the drug was excreted both via glomerular filtration and active tubular secretion. It was observed that only 72.4% of the drug was detectable in the urine without any of its metabolites [45,62,63], and this suggested that additional mechanisms were required for its excretion. Animal experiments described in Beckmann et al. [64] and Yoh et al. [67] highlighted the presence of buformin in the bile and the transport of this biguanide from the blood to the intestinal lumen. Lintz et al. confirmed this data in patients by detecting radioactive signals in the intestinal fluid after intravenous administration of butylbiguanide [66]. However, it was not clear if the drug could reach the intestinal lumen via the bile or via the intestinal mucosa.

In additional studies, five fasted diabetic patients received 100 mg micronized ^14^C-butylbiguanide (50 IxCi) in hard-shell capsules orally. Mean plasma concentration after 1 h from administration extrapolated from their report was 4.33 μM [66]. An average of 74.4% of the amount of drug administered was excreted by the kidneys of the 4 test subjects, whereas a higher value had been found in previous investigations [45,62,64,68].

After oral administration of butylbiguanide, high concentrations of the drug were detected in intestinal fluid, with a maximum value of 700 μg/mL. The concentration of butylbiguanide in the intestinal fluid of the jejunum 4–5 h after oral administration was still significantly higher than the amount detected after intravenous administration, and remained almost constant for a long period, indicating that there was a significant accumulation of butylbiguanide in the intestinal mucosa, which was more significant after oral administration rather than intravenous administration. After intravenous injection, the concentration of butylbiguanide in the intestinal epithelium was 6–11 times higher than in plasma, and after oral administration, it was 10–35 times higher than in plasma, with only one exception. Accumulation of butylbiguanide in the intestinal mucosa in humans corresponds to that found in animal studies [66,67,69,70]. For example, 3 h after oral administration of 10 mg/kg butylbiguanide, a concentration of 13 μg/g was found in the intestinal wall of rats, while in plasma concentration was 5.72 μM [69]. After intravenous administration of 50 mg butylbiguanide, its concentration in the liver was 12.72–25.44 μM [66]. The accumulation was even greater after oral administration: in two patients, 2–3 h after oral administration, the detected liver concentrations were 63.61–127.21 μM [66].

**Table 1:** Therapeutic concentrations of biguanides.

No.	Drug	Dosage	Mean of Administration	Concentration	Treatment Duration	Model	References
1	Metformin	1.5–2.5 g/day	Oral	4–15 μM	1.5–3 h	Human	[10]
2	Metformin	1.7/2.55 g/day	Oral	3.8 μM	0.3–2.5 h	Human	[26]
3	Metformin	1 g	Oral	14–22 μM	3 h	Human	[27]
4	Metformin	0.85–1.70 g/day	Oral	250 μmol/Kg–4 mmol/Kg	2–3/3V5 weeks	Human	[28]
5	Metformin	0.5 g	Intravenous	11.6–38.68 μM	1–2 h	Human	[29]
6	Metformin	0.35 g/Kg	Oral	200–1500 μM	3 weeks	Mouse	[22]
7	Metformin	0.35 g/Kg	Intraperitoneal	7.5–100 μM	2 weeks	Mouse	[22]
8	Metformin	0.125 g/Kg	Intraperitoneal	42–184 μM	0.5 h	Mouse	[31]
9	Metformin	0.05 g/Kg	Oral	147–1206 μM	0.5 h	Mouse	[32]
10	Metformin	0.05 g/Kg	Intravenous	38–55 μM	0.5 h	Mouse	[32]
11	Phenformin	0.1 g	Oral	0.97 μM	2 h	Human	[45]
12	Phenformin	0.1 g	Oral	0.72 μM	3 h	Human	[48]
13	Phenformin	0.05 g	Intravenous	0.19 μM	3.5 h	Human	[49]
14	Phenformin	66 ± 20 mg/day	Oral	0.14–0.56 μM	5 ± 3 years	Human	[50]
15	Phenformin	0.15 g/day	Oral	0.5–1.17 μM	N/A	Human	[51]
16	Phenformin	0.3 g/Kg/day	Oral	1.4 μM	10 days	Mouse	[52]
17	Phenformin	0.0125 g/Kg	Intravenous	3.4 μM	0.5 h	Mouse	[52]
18	Phenformin	1.8 mg/mL	Oral	1–1.5 μM	5–7 days	Mouse	[54]
19	Phenformin	0.2 g/Kg	Oral	1.49 μM	28 days	Mouse	[55]
20	Phenformin	0.1 g/Kg	Oral	2–3.1 mM	1–2 h	Mouse	[56]
21	Phenformin	0.1 g/Kg	Intraperitoneal	0.9–2.6 mM	1–2 h	Mouse	[56]
22	Phenformin	0.05 g/Kg	Oral	2.5 μM–3.49 μM	0.5–4 h	Rat	[57]
23	Phenformin	1.5 g	Oral	1.17 μM	N/A	Human	[58]
24	Buformin	0.05 g	Intravenous	0.45–2.13 μM	1–5 h	Human	[66]
25	Buformin	0.1 g	Oral	4.33 μM	1 h	Human	[66]
26	Buformin	0.01 g/Kg	Oral	5.72 μM	3 h	Rat	[69]
27	Buformin	0.05g	Intravenous	12.72–25.44 μM	2–3 h	Human	[66]
28	Buformin	0.1g	Oral	63.61–127.21 μM	2–3 h	Human	[66]

## 3. The Mechanism of Action of Biguanides: Lessons from Type 2 Diabetes Mellitus (T2DM)

Type 2 diabetes mellitus (T2DM) is the most common type of diabetes observed in the population and a leading cause of death [71]. T2DM is characterized by insulin resistance, βcell dysfunction, and elevated hepatic glucose output mainly attributed to an increase in gluconeogenesis [72,73]. 

Biguanides have been used for the treatment of type 2 diabetes mellitus (T2DM) for more than 70 years and metformin is the most prescribed oral anti-diabetic agent worldwide, taken by over 150 million people annually [74]. Metformin prevents body weight gain and does not cause hypoglycemia, which is frequently associated with the use of other antidiabetic drugs [75]. Moreover, metformin may have therapeutic potential in the treatment of conditions such as nephropathy [76], polycystic ovary syndrome [77], and cardiovascular diseases [78,79], often associated with diabetes or insulin resistance.

The pleiotropic properties of metformin suggest that the drug acts on multiple tissues, but the underlying mechanism of action remains debated.

Most of the studies on the mechanism of action of biguanides, especially metformin, have been conducted in T2DM models, trying to identify the primary target and the consequences of its alteration. These studies have then ignited investigation in tumor models, to determine if the effectors and mechanisms operating in diabetes could also be responsible for the antitumor properties of these drugs.

The main and best studied site of the antidiabetic action of biguanides is the liver, where these drugs reduce hepatic gluconeogenesis, through various mechanisms discussed below. However, other studies have also proposed the gut and skeletal muscle as additional sites responsible for the blood-glucose-lowering properties of biguanides.

### 3.1. Liver as a Target Tissue

A clinical study using ^13^C nuclear magnetic resonance spectroscopy showed that metformin reduces fasting plasma glucose concentrations in diabetic patients by decreasing hepatic glucose production (HGP) by about 25% and gluconeogenesis (two to three times higher in diabetics than in control patients) by about 35%, without affecting glycogenolysis [80].

Several mechanisms have been identified for the action of biguanides in hepatic gluconeogenesis and glucose production, which are generally thought to be mediated by the interaction of the drugs with two main cell compartments: mitochondria (energy or redox alterations) or lysosomes.

#### 3.1.1. Energy-Dependent Mechanisms: The Controversial Role of the Complex I—AMPK Axis

In 2000, two independent groups reported for the first time that metformin inhibits the mitochondrial respiratory chain complex I thus decreasing NADH oxidation, proton pumping across the inner mitochondrial membrane, and oxygen consumption rate [81,82].

The mammalian mitochondrial respiratory complex I, also known as NADH-ubiquinone oxidoreductase, is a large L-shaped membrane-bound enzyme consisting of many core and accessory subunits, that oxidizes NADH to NAD^+^ and transfers four protons from the mitochondrial matrix to the transmembrane space and electrons to the ubiquinone pool [83].

**Figure 1:**
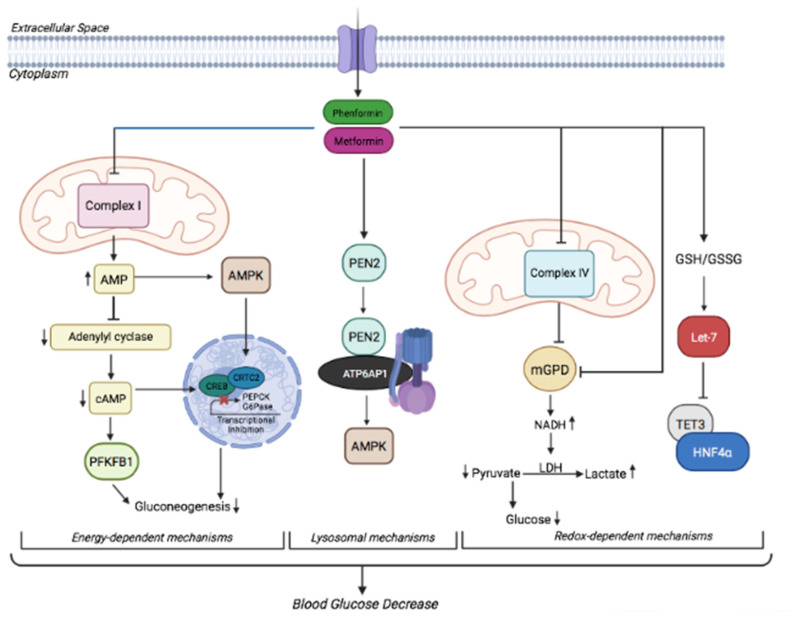
Proposed mechanisms for the glucose-lowering properties of biguanides. (Left) Energy-dependent mechanisms. Supra-pharmacological concentrations of biguanides suppress glucose production through the inhibition of complex I, which leads to the activation of AMPK and inhibition of the cAMP-PKA pathway. (Middle) Lysosomal mechanisms. Pharmacological concentrations activate PEN2, which inhibits lysosomal v-ATPase and activates AMPK in the intestine, decreasing blood glucose levels. (Right) Redox-dependent mechanisms. Biguanides inhibit mitochondrial complex IV, which results in inhibition of mitochondrial glycerol 3-phosphate dehydrogenase (mGPD) activity and gluconeogenic program. Alternatively, pharmacologic biguanides concentrations directly inhibit mGPD, leading to an increase in cytosolic NADH levels, which prevents lactate utilization and decreases hepatic glucose output. On the other hand, clinically relevant concentrations of biguanides up-regulate microRNA let-7, leading to the downregulation of TET3 and changes in the ratio of HNF4α isoforms, with consequent gluconeogenesis inhibition.

The molecular interaction mechanism between biguanides and the mitochondrial respiratory chain complex I has not been completely understood (Figure 1). A proposed mechanism suggests that metformin binds the Cys-39 in the amphipathic region at the interface of the hydrophilic and membrane domains, trapping the enzyme in a deactive-like open-loop conformation [84]. Complex I inhibition causes a decline in intracellular ATP levels concomitantly with an increase in intracellular ADP and AMP. This altered cellular energy charge activates the energy sensor AMPK [85], already reported to be activated by metformin in 2001 [86]. These two seminal discoveries, the decrease of energy metabolism and activation of AMPK, were at the center of the proposed mechanism of action of biguanides for the following years. In 2005, Shaw and colleagues showed that metformin requires LKB1, a kinase that phosphorylates and activates AMPK, to lower blood glucose levels in the liver of adult mice. Loss of LKB1 increased gluconeogenesis and abolished metformin glucose-lowering activity [87]. Once activated by LKB1, AMPK phosphorylates TORC2/CRTC2, the CREB (cAMP response element-binding protein) transcriptional coactivator, and sequesters this factor into the cytoplasm, preventing PPARγ coactivator 1α (PGC1α) transcription and subsequent increase of gluconeogenic phosphoenolpyruvate carboxylase (PEPCK) and glucose-6-phosphatase (G6Pase) target gene expression [87]. A few years later, this mechanism of action was challenged by the evidence that, in response to metformin administration, blood glucose levels, hepatocytes glucose production, and gluconeogenic gene expression were not changed in mice lacking AMPK in the liver, compared to wild-type littermates. Moreover, the metformin glucose-lowering effect was maintained even under forced expression of gluconeogenic genes through PGC-1α overexpression [88]. Thus, metformin inhibited gluconeogenesis independently of LKB1/AMPK.

The gluconeogenic pathway is a high-energy-consuming process that requires six ATP equivalents for each molecule of glucose produced. Since AMP is a potent allosteric inhibitor of fructose 1,6-bisphosphatase (FBP1), a key enzyme in gluconeogenesis, it was proposed that by raising AMP levels metformin inhibits gluconeogenesis through FBP1 inhibition. Supporting this hypothesis, a point mutation in FBP1 that renders the enzyme insensitive to AMP was found to abrogate the response to metformin in vivo [89]. A further breakthrough study in 2013 showed a novel mechanism of action for biguanides-driven hypoglycemic function independent of AMPK [90] whereby biguanides were suggested to antagonize the action of glucagon by inhibiting the activity of the cAMP-activated protein kinase A (PKA). Through their effect on complex I and consequent accumulation of cellular AMP, biguanides inhibit adenylate cyclase and reduce the levels of cyclic AMP, abrogating the phosphorylation of critical PKA substrates, including the 6-phosphofructo-2-kinase isoform 1 (PFKFB1). Phosphorylation of PFKFB1 inhibits the formation of fructose-2,6-bisphosphate, an intracellular mediator that acutely activates the glycolytic enzyme 6-phosphofructo-1-kinase and inhibits the gluconeogenic enzyme fructose-1,6-bisphosphatase. Lowering of cAMP would therefore inhibit the switch from glycolysis to gluconeogenesis triggered by glucagon [91]. Hence, according to these studies, the metformin-driven complex I inhibition, and consequent decrease of ATP/AMP ratio, could block the gluconeogenic flux independently of AMPK. Other studies added further evidence arguing against the involvement of AMPK in hepatic glucose production. Using liver-specific AMPK knock-out mice, Hasenour and colleagues showed that AMPK is not required for suppression of hepatic glucose production induced by AICAR, an inducer of metabolic stress [92]. More recently, Cokorinos et al. showed that a non-selective AMPK agonist lowered blood glucose levels by inducing an AMPK-mediated increase of glucose disposal in skeletal muscle, without inhibiting hepatic glucose production [93].

In addition to the growing skepticism about the involvement of AMPK in the inhibition of gluconeogenesis in response to metformin, in more recent years, some researchers started also being concerned that only supra-physiological concentrations of biguanides could directly inhibit mitochondrial complex I activity [74]. In isolated mitochondria or in sub-mitochondrial particles, concentrations of metformin between 20 and 100 mM are required for complex I inhibition [94], and the half-maximal inhibitory concentration (IC50) for complex I inhibition is reported to fall within the micromolar range (~500 μM) for phenformin [84]. Furthermore, it has been reported that the concentration of metformin required to inhibit complex I is lower in intact cells than in isolated mitochondria. An explanation that was proposed to solve this discrepancy was that metformin accumulates in the mitochondria in a voltage-dependent manner, reaching millimolar concentrations compared to the micromolar concentrations in the cytosol [95]. However, many authors argue against the hypothesis that metformin accumulates in the mitochondria. Indeed, a major concern is that the mitochondrial inner membrane allows the passage of hydrophilic molecules only through specific transporters but there is no evidence that supports the existence of a carrier specific for metformin. Moreover, the entrance of numerous positive charges in the mitochondria is expected to cause a collapse of mitochondrial membrane potential, while some authors show that metformin is not able to depolarize isolated mitochondria [95,96].

Defects in mitochondrial respiratory chain activity are reported to contribute to the development of insulin resistance and hyperglycemia in T2DM [97,98,99,100]. Mitochondria have a peculiar life cycle that includes continuous phases of fusion and fission necessary for the maintenance of their bioenergetic efficiency [101,102]. Impairing these mechanisms leads to defects of the mitochondrial functions and culminates in the decrease of mitochondrial respiration [103,104]. Wang et al. show that micromolar concentrations of metformin (75 μM) not only fail to inhibit complex I activity but also improve mitochondrial respiration by increasing mitochondrial fission through AMPK signaling. The authors suggest that the decrease in ATP levels and oxygen consumption rate observed with supra-pharmacological doses of metformin would rather be a consequence of adenine synthesis inhibition. Insufficient levels of cellular ADP would lead to an inability to utilize the mitochondrial membrane potential to generate ATP. To support this hypothesis, they showed that the enzymatic activity of purified mitochondrial complexes is unchanged after metformin treatment at all concentrations, including 1000 μM [105]. Accordingly, using permeabilized skeletal muscles derived from type II diabetes patients, Larsen and colleagues tested a wide range of metformin concentrations revealing that the minimum concentration needed to appreciate a significant reduction of complex I activity is 3 mM [106].

#### 3.1.2. Redox-Dependent Mechanisms

In an attempt to address the concerns about the dosage, Madiraju and colleagues showed that by administering to rats doses of metformin corresponding to the range used in T2DM patients (20–50 mg/Kg), metformin increased hepatic cytosolic NADH/NAD^+^ ratio to impair glucose production from redox-dependent substrates (lactate and glycerol), independently of complex I [107]. The authors proposed that this redox alteration is due to inhibition of the mitochondrial glycerol-3-phosphate dehydrogenase (mGPD) activity, a key component of the glycerophosphate shuttle (GPS), which is one of two shuttle systems required to transfer reducing equivalents from the cytosol to the mitochondria (Figure 1). mGPD is localized in the outer face of the inner mitochondrial membrane and oxidizes glycerol-3-phosphate (G3P) to dihydroxyacetone phosphate (DAP) with concurrent reduction of flavin adenine dinucleotide (FAD) to FADH2. Its cytosolic partner cGPD reduces DAP to G3P while oxidizing cytosolic NADH [108].

Acute and chronic metformin treatment elicited a significant decrease in the mitochondrial redox state and an increase in the cytosolic redox state, impairing glucose production from lactate. Furthermore, mGPD knockdown phenocopied metformin activity in vivo and abolished metformin effects [107]. In a further study, the same group showed that metformin inhibits hepatic gluconeogenesis in a redox-dependent manner without affecting mitochondrial citrate synthase flux and hepatic energy charge [27]. They infused awake rats with ^13^C-labeled lactate or alanine and traced these molecules through the gluconeogenic flux using ^13^C NMR spectroscopy, finding that metformin impedes the hepatic conversion of reduced substrates (lactate and glycerol), but not oxidized substrates (alanine and pyruvate) into glucose [27].

These observations provided a plausible explanation for the mechanism of action of biguanides at therapeutic doses, although they also raised some criticisms. A first concern regards the role of glycerophosphate shuttle in the liver since it is less relevant than the malate-aspartate shuttle (MAS), the other NADH shuttle. Thus, glycerol-phosphate shuttle (GPS) inhibition may not be sufficient to prevent gluconeogenesis [109]. Indeed, mice with selective disruption of the glycerol–phosphate shuttle showed unchanged fasting blood glucose levels, while knockout of malate–aspartate shuttles resulted in a significant decrease of blood glucose levels that was further reduced in mice with double inactivation of GPS and MAS [110]. Alshawi and coll. [111] found that a low dose of metformin (<2 nmol/mg) caused a more oxidized mitochondrial NADH/NAD^+^ state and an increase in lactate/pyruvate ratio, supporting previous findings by Madiraju et al. However, in contrast to these authors, they found that metformin prevented gluconeogenesis from both reduced and oxidized substrates and did not inhibit mGPD activity. Instead, they found that metformin accumulates in the mitochondria due to its positive charge, depolarizing the mitochondrial membrane and causing inhibition of citrin, the electrogenic transporter for aspartate, and consequent inhibition of the malate-aspartate shuttle. To compensate for this inhibition, the glycerol-phosphate shuttle is stimulated and leads to a decrease of glycerol-3-phosphate, a potent allosteric inhibitor of phosphofructokinase 1 (PFK1). As a result, decreased G3P stimulates PFK1 and glycolysis and inhibits gluconeogenesis. However, the lack of inhibition by metformin on malate dehydrogenase or aspartate aminotransferase observed by Madiraju et al. [107], argues against this interpretation.

Calza et al. failed to observe a reduction of lactate-induced hepatic glucose output by metformin in rats [112] and MacDonald et al. did not see direct inhibition of mGPD by metformin in biochemical assays [113].

In a very recent publication, LaMoia et al. provided novel evidence to resolve these controversies, supporting mGPD, but not complex I inhibition as a major determinant of metformin inhibition of hepatic gluconeogenesis [114]. 

They demonstrated that biguanides (metformin, phenformin, galegine) repress hepatic gluconeogenesis from the redox-dependent substrate glycerol by blocking complex IV, which in turn results in inhibition of mGPD activity and increased cytosolic redox state. Inhibition of complex IV was proposed to backlog the electron transport chain (ETC) and cause indirect mGPD inhibition. Conversely, the authors showed that the specific complex I inhibitor piericidin A was unable to prevent gluconeogenesis from glycerol, while the specific complex IV inhibitor KCN phenocopied the effect of biguanides in vitro.

While the issue that mGPD is a direct target of biguanides needs to be properly addressed with compelling biochemical approaches, the authors noted that most of the biochemical assays arguing against mGPD were performed using KCN or other complex IV inhibitors in the reaction buffer. Hence, considering this new finding, it is possible that these inhibitors may have masked the effect of biguanides on GPD2 activity [114]. 

In another recent article, metformin administered at clinically relevant concentrations was shown to inhibit gluconeogenesis in primary hepatocytes and animal models of type 2 diabetes by activating the let-7/TET3/HNF4α axis in a redox-dependent fashion [115]. They demonstrated that clinically relevant doses of metformin up-regulate microRNA let-7, leading to the downregulation of TET3 and changes in the ratio of HNF4α isoforms, with consequent transcriptional inhibition of the gluconeogenic gene program (Figure 1). Therefore, these observations further support the modulation of the redox state as a determinant of metformin inhibition of hepatic gluconeogenesis.

#### 3.1.3. Lysosomal Mechanisms

Very recently, Ma and colleagues have proposed a further alternative mechanism, whereby low doses of metformin activate AMPK by inhibiting lysosomal v-ATPase, independently of energy charge [23]. Previous observations from the same group demonstrated that AMPK could be activated by low glucose through aldolase, which senses the decrease of fructose-1,6-biphosphate FBP and forms a complex with v-ATPase, Regulator, axin, LKB1 that activates AMPK [116]. Hence, low glucose activates AMPK independently of ATP/AMP ratio, by regulating lysosomal v-ATPase. By performing a proteomic screening of metformin-interacting lysosomal proteins with a biotinylated photoactive probe, the authors identified PEN2 as a direct metformin interacting protein and found that, after binding with the drug, PEN2 associates with ATP6AP1, a member of the v-ATPase complex, thereby causing inhibition of the ATPase complex and activation of AMPK (Figure 1). Of note, loss of hepatic PEN2 abrogated the ability of metformin to lower hepatocyte fat content in mice, while conditional PEN2 knockout in the gut abrogated its glucose-lowering effect.

Together, these data support the idea that AMPK activation by this lysosomal-mediated mechanism is responsible for the therapeutic action of metformin. However, since other studies failed to detect phosphorylation of the AMPK substrate ACC in the liver following metformin administration in mice [27], this novel mechanism requires further investigation.

### 3.2. Gut as a Target Tissue

Biguanides accumulate in the small intestine at concentrations that are up to 20–300 times greater than plasma [32], suggesting that the gut could be an important site for biguanides action.

Early studies provided evidence that intravenous injection of metformin did not significantly lower glucose levels [117,118], although only acute effects were evaluated in those reports. Also, an increase in metformin concentration in plasma through inhibition of the MATE transporter, which mediates hepatic and renal elimination of the drug, had little effect on circulating glucose levels [119]. Furthermore, a gut-restricted formulation of metformin had greater glucose-lowering efficacy than systemically absorbed formulation [120]. These observations have been linked to a reduction in the rate of glucose absorption in the small intestine [121] and an increase in glucose uptake from the bloodstream and its utilization in metformin-treated enterocytes. Two different studies measured glucose uptake in diabetic patients or healthy volunteers treated with metformin using [18F]-fluoro-2-deoxy-D-glucose (FDG), a non-metabolized glucose analog. PET-computed tomography revealed a three-fold increase in FDG uptake in the small intestine and especially in the colon [122,123]. 

In addition to the increased glucose uptake and utilization in the enterocytes, in recent years the mechanism of biguanides action in the gut has been also linked to their ability to alter the secretion of some key molecules (GLP1 and GDF15) or to affect the composition of the gut microbiota.

#### 3.2.1. Glp-1

Glucagon-like peptide 1 (GLP1) is an incretin hormone secreted from the intestinal enteroendocrine L cells in response to the presence of nutrients in the intestinal lumen. In healthy individuals, incretins are responsible for up to 70% of insulin secretion after an oral glucose load and their effect is severely impaired in T2DM patients [124]. GLP1 is essential for glucose homeostasis acting through a gut-brain neuronal axis that provides insulin secretion, inhibition of glucagon secretion, slowing of gastric emptying, and a reduction in appetite and food intake.

According to recent studies, metformin may increase the secretion of GLP1 from enteroendocrine L cells by direct and indirect mechanisms and may induce the expression of the GLP1 receptor [125].

In a double-blinded randomized placebo-controlled trial, healthy patients showed an overall increase of 23.4% of GLP1 plasma concentration after treatment with metformin for 18 months compared to placebo [126]. Another landmark study demonstrated that 75% of acute glucose-lowering properties of metformin could be attributed to its direct stimulation of GLP-1 from L cells and that a GLP1 receptor antagonist could prevent the observed decrease of blood glucose [127]. Conversely, other studies demonstrate an indirect effect of metformin on GLP1 levels through the modulation of dipeptidyl peptidase-4 (DPP4) [128], while other authors did not observe any effect on DPP4 [129]. Hence, the actual mechanism and involvement of GLP1 signaling in the response to biguanides are still unclear and need to be further clarified.

#### 3.2.2. Gdf-15

Obesity is one of the main risk factors for T2DM and people with type 2 diabetes show a significant metformin-induced body weight loss [130,131]. This effect has been recently linked to an increased secretion of growth differentiation factor 15 (GDF15) [132,133]. 

GDF15 is a divergent TGF-β superfamily cytokine that acts through the recently identified orphan receptor GFRAL (GDNF receptor α-like), a member of the glial-cell-derived neurotropic factor family (GDNF), which is expressed in the area postrema in the brainstem of mice, rats, monkeys, and humans [134]. 

In 2006, it was observed that transgenic mice with ubiquitous expression of the full-length human GDF15 protein showed a significant reduction in body weight compared to non-transgenic littermates [135]. Despite equivalent food intake, transgenic GDF15 mice had less white and brown fat, improved glucose tolerance, lower insulin levels, and were resistant to dietary-and genetic-induced obesity [136]. In wild-type mice, oral metformin increased GDF15 circulating protein levels and GDF15 mRNA in the small intestine, colon, and kidney. Metformin decreased food intake and prevented weight gain in response to a high-fat diet in wild-type mice but not in mice lacking GDF15 or its receptor. In obese mice on a high-fat diet, the effects of metformin to reduce body weight were reversed by a GFRAL-antagonist antibody [132], suggesting that metformin activity could be mediated by GDF-15.

GDF15 is also essential for the increased insulin sensitivity associated with the use of metformin. The pharmacological mechanism underlying the metformin induction of GDF15 seems to involve the integrated stress pathway [132]. In primary mouse hepatocytes, metformin stimulates the secretion of GDF15 by increasing the expression of activating transcription factor 4 (ATF4) and C/EBP homologous protein (CHOP) [133]. The new insight that the lower small intestine and colon are major sites of metformin-induced GDF15 expression, provides further evidence that metformin can mediate its benefits, at least in part, by acting on the intestinal epithelium as a major target.

#### 3.2.3. Gut Microbiota

High interest has been focused on the gut microbiota as a target of metformin action. A double-blind study indicated that metformin can change intestinal microbiota composition in human patients and that glucose tolerance is improved in mice receiving metformin-altered microbiota [121]. Metagenomic and metabolomic analysis of samples from individuals with T2DM and treated with metformin for 3 days, revealed that metformin treatment increased the levels of the bile acid glycoursodeoxycholic acid (GUDCA) in the gut by decreasing the abundance of species of *Bacteroides fragilis*. It was found that GUDCA is a novel antagonist of intestinal FXR, a ligand-activated nuclear receptor that regulates hepatic bile acid biosynthesis, transport, and secretion and may inhibit GLP1 secretion from L cells [137]. In addition, metformin increases the abundance of short-chain fatty acid (SCFA)-producing bacteria and facilitates SCFA-induced GLP1 secretion via signaling through GPR41 and GPR43 in L cells [138]. However, in contrast with all these observations, a different study showed that metformin significantly improved oral glucose tolerance also in GLP1R^−/−^ mice and in wild-type mice fed with a high-fat diet and treated with a GLP1R inhibitor [125].

### 3.3. Muscle as a Target Tissue

Some studies have suggested that skeletal muscle may be involved in the glucose-lowering properties of metformin. Early studies showed that metformin lowers glucose levels in T2DM patients by increasing insulin-stimulated glucose uptake [80,139,140]. 

In isolated skeletal muscle, Zhou et al. reported that metformin activated AMPK and concomitantly increased glucose uptake, an effect that was additive with insulin stimulation [86]. These observations led to the conclusion that, by inhibiting complex I and activating AMPK, metformin promotes glucose uptake in muscle [93] and enhances insulin sensitivity [141]. However, this hypothesis has been challenged by a very recent study on the muscle-specific knockout of AMPKα1/α2 mouse models, where it was shown that lack of AMPK activity in skeletal muscle of lean and diet-induced obese mice does not affect the ability of metformin to lower blood glucose levels or improve whole-body glucose tolerance [142]. Moreover, in T2DM patients rendered normoglycemic with 4 weeks of insulin treatment, metformin had no effect on insulin-stimulated peripheral glucose metabolism [143], suggesting that the ability of metformin to increase insulin-stimulated muscle glucose uptake could be secondary to improved glucose homeostasis and reduction of glucose toxicity rather than due to a direct effect.

## 4. Biguanides and Cancer

The anti-tumor properties of biguanides were unknown until 2005, when Evans et al. [144] identified in diabetic patients an inverse correlation between metformin treatment and cancer occurrence, paving the way for the exploration of biguanides usage in cancer therapy and prevention. Until December 2021, metformin has been investigated in 1901 clinical trials on various types of cancer, and 216 of them are still underway. While studies seem to support the anti-tumor effects of metformin in diabetic patients, less is known about the therapeutic effect of metformin in non-diabetic cancer patients. Many studies have been focused on the understanding of the molecular mechanism underlying the anti-cancer properties of biguanides that led to the identification of a plethora of different molecular targets. Similar to the research on diabetes, in this context, the exact mechanism by which biguanides operate and their target selectivity in different experimental conditions is still controversial, due to the lack of a unifying model.

In general, biguanides are believed to exert their antitumor properties by two main mechanisms: direct, by acting directly on the tumor cells and inhibiting their growth, and indirect, by inducing changes in the body that ultimately affect tumorigenesis.

### 4.1. Direct Antitumor Effects

The notion that biguanides exert direct antitumor effects comes mostly from the evidence that the growth, proliferation, viability, and/or motility of cultured cancer cells are impaired upon exposure to the drugs. As for the regulation of glucose homeostasis, also in this context, mitochondria are believed to be the main site of biguanides action, and AMPK is a critical mediator of their therapeutic effects.

#### 4.1.1. Mitochondrial Mechanisms

**Figure 2:**
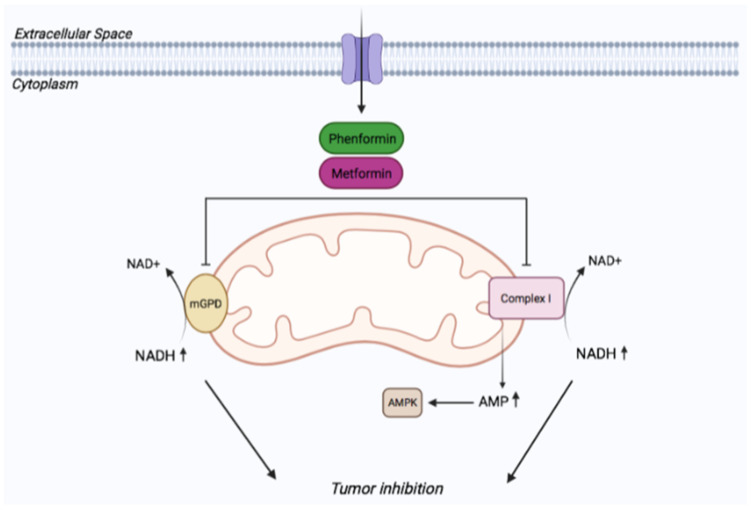
Redox-dependent inhibition of tumor growth by biguanides. Therapeutic doses of biguanides inhibit mGPD in cancer cells, increasing NADH content and redox state and inhibiting tumor growth. Supra-pharmacologic concentrations of biguanides inhibit complex I, increasing NADH content and AMP levels and suppressing tumor growth.

Most studies addressing the mechanism of action of biguanides have been focused on targets localized into the mitochondria (Figure 2). As discussed above, it is widely recognized that metformin is capable of inhibiting complex I of the electron transport chain. Supporting the role of complex I inhibition as an important player in the anti-tumorigenic effect of metformin target in cancer, cells expressing the rotenone-resistant yeast complex I analog NDI1 were no longer inhibited by metformin [145]. Similarly, ectopic expression of NDI1 impaired the ability of phenformin to inhibit cancer cell proliferation and oxygen consumption, although only in cells with complex I mutations [146]. However, while the use of NDI1 overexpression is generally considered relevant evidence to confirm complex I involvement, it has to be noted that NDI1 corrects the NAD^+^/NADH ratio, which can be reduced by many alterations in mitochondria other than inhibition of complex I (e.g., see [147]). 

Also, the use of NDI1 may have limitations if not carefully controlled. For instance, its exclusive localization in the mitochondria should be verified, the expression levels should be monitored during experimentation, and complex I should be inactivated in cells expressing NDI1, to avoid artifactual results.

Targeting complex I using small molecules has shown anti-cancer efficacy in vitro and in animal models [148,149]. Several observations point to the inhibition of complex I as the main mechanism of action of metformin in cancer cells. In human oral squamous carcinoma KB cells, metformin (0.1–10 mM) specifically inhibits complex I, both in intact cells and after permeabilization [150]. Metformin (3–10 mM) effectively diminished pancreatic cancer stem cells by the inhibition of mitochondrial respiration [151]. In permeabilized human HCT116 p53^-/-^ colorectal carcinoma cells expressing NDI1, metformin (0.25–1 mM) failed to decrease cell proliferation [145], while metformin (1–10 mM) potently inhibited mitochondrial complex I in pancreatic ductal adenocarcinoma cells [152]. 

In 2019, Momcilovic and colleagues used 4-(18F) fluorobenzyl-triphenylphosphonium (18F-BnTP PET) imaging to detect in vivo changes in mitochondrial membrane potential in a mouse model of lung cancer. They showed that phenformin decreases the uptake of the tracer, indicating the ability of the drug to lower mitochondrial membrane potential (ψ), a consequence attributed by the authors to complex I inhibition [153], although a decrease of membrane potential can also be caused by inhibition of other mitochondrial targets, such as mGPD [154] or by the accumulation of the positively charged biguanide in the mitochondria.

Since in the majority of the above-mentioned studies biguanides have been used at supraphysiological doses that are unlikely to reflect the actual concentrations measured in humans and animal models [33,155,156], it is generally tempted to believe that other mechanisms, beyond complex I inhibition, may operate on cellular and animal models exposed to therapeutic concentrations of biguanides.

Recent work carried out on Sonic Hedgehog-driven medulloblastoma cells showed that pharmacological phenformin concentrations (1–5 μM) inhibit tumor growth independently of complex I and AMPK, through alterations in cytoplasmic redox potential and increased NADH levels [52], by inhibiting glycerol-3-phosphate dehydrogenase. Elevated NADH levels promote the association between the redox sensor CtBP2 and the transcription factor GLI1, leading to inhibition of Hedgehog-dependent transcriptional output and medulloblastoma growth.

In keeping with these findings, it has been observed that in thyroid cancer cells, metformin inhibits the activity and downregulates the expression of mGPD, decreasing their growth and metabolism [157]. Another work showed that low expression of cGPD correlates with poor responses to metformin in 15 cell lines of various cancer types and that cGPD overexpression enhanced the anticancer activity of metformin, leading to glycerol-3-phosphate overproduction and inhibition of mitochondrial function [158]. 

In contrast to this study, it was shown that ablation of cGPD enhanced the inhibition of tumor growth mediated by metformin, although the biguanide was given at supraphysiological concentrations [159].

Consistent with redox imbalance as a major alteration underlying the antiproliferative effect of metformin, Gui and collaborators [160] proposed that metformin’s anti-proliferative effect is due to loss of NAD^+^/NADH homeostasis and inhibition of aspartate biosynthesis, an effect that was attributed to the blockade of NADH dehydrogenase activity of complex I rather than to mGPD inhibition and that could be rescued by pyruvate, due to its ability to regenerate NAD^+^.

Therefore, these latter studies seem to point at NADH/NAD^+^ alteration as key mechanisms underlying the antitumor properties of biguanides, although concerns about the primary target need to be properly addressed, as discussed above (Figure 2).

#### 4.1.2. AMPK as a Mediator of the Response to Biguanides in Cancer

Although the activation of the energy sensor AMPK represents one of the most frequently evoked events accompanying biguanides therapeutic action, the role of AMPK in cancer seems to be ambiguous [161]. The discovery that AMPK is the key downstream effector of the tumor suppressor LKB1 and the ability of AMPK to inhibit fatty acid synthesis, mRNA translation, and cell growth support the notion that this kinase acts as a tumor suppressor. However, in different contexts, at different stages of tumor development or under certain conditions (e.g., metabolic stress), AMPK seems to function as a tumor promoter, by activating programs that facilitate cancer progression and survival [162]. 

In this view, the use of AMPK agonists is now suggested to be more appropriate for cancer prevention, while AMPK inhibitors seem to be better suited for the treatment of established malignancies [161]. 

Supporting the notion of a tumor-promoting function of AMPK, phenformin was shown to be more effective in reducing lung tumor growth when cells lacked a functional LKB1/AMPK pathway [54].

**Figure 3:**
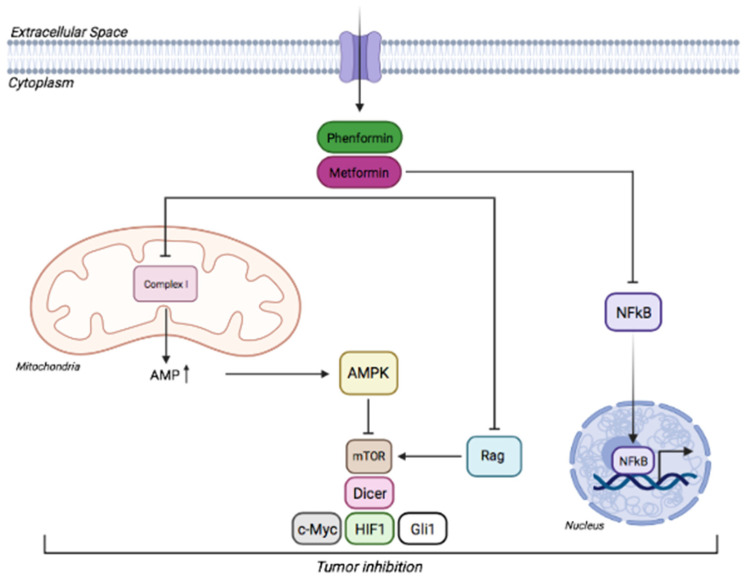
AMPK-dependent and AMPK-independent inhibition of tumor growth by biguanides. Supra-pharmacological concentrations of biguanides inhibit complex I, which increases AMP levels and leads to the activation of AMPK. Alternatively, metformin prevents the activation of NFkB pathway by inhibiting the translocation of NFkB to the nucleus. AMPK regulates DICER, cMyc, HIF1α, and Gli1 activity and inhibits mTOR complex, suppressing tumor growth. Biguanides also inhibit Rag GTPases to suppress mTOR signaling.

However, many studies have supported the metformin-mediated activation of AMPK as a tumor-suppressive mechanism (Figure 3). In the “classical” mechanism, metformin inhibits complex I of the mitochondrial respiratory chain and ATP synthase, raising the levels of intracellular AMP/ADP that trigger the activation of AMPK [41]. Alternatively, metformin may activate AMPK through the lysosomal pathway by a non-canonical mechanism [163]. Indeed, AMPK can be activated by low concentrations of metformin through the formation of a complex with Axin and late endosomal/lysosomal adaptor, MAPK, and LAMTOR1. Thus, metformin might also activate AMPK by a mechanism involving the lysosomes, rather than complex I.

Once activated, AMPK is thought to inhibit key substrates involved in cell growth and proliferation, being the most relevant and best-studied the mechanistic Target Of Rapamycin Complex 1 (mTORC1). mTORC1 plays a key role in controlling the metabolism, growth, and proliferation of cancer cells [164,165] mostly by phosphorylating two key targets: S6 Kinase 1 (S6K1) and initiation factor 4E binding protein 1 (4E-BP1) [166,167]. By activating AMPK, biguanides are thought to inhibit mTORC1 through phosphorylation of TSC1, TSC2, and Raptor [168,169]. Additionally, Kalender and collaborators demonstrated that biguanides suppress mTORC1 signaling also independently of AMPK and TSC1/2, by inhibiting Rag GTPases [170].

Besides mTORC1 inhibition, AMPK has been also shown to promote p53 activation via phosphorylation of Ser15, thus promoting cell survival in response to glucose limitation [171] and p53-deficient cancer cells were shown to be more sensitive to metformin treatment [172], indicating that p53 regulates cancer cells survival in response to metformin-induced metabolic changes.

Other targets regulated by metformin via AMPK, causing inhibition of cancer cell proliferation by blocking the Warburg effect are DICER, cMyc, HIF1α [173]. Conversely, other works found that metformin inhibits the growth of various cancers by preventing nuclear translocation of the transcription factor NFkB, an effect that was believed to be independent of AMPK [174,175,176,177] (Figure 3).

In a work on ovarian cancer patients, it was shown that metformin treatment affects pathways related to mitochondrial metabolism involving nucleotide metabolism, redox, and energy status [178]. More recently, a study in breast cancer patients showed that metformin reduces the levels of mitochondrial metabolites and increases 18-FDG flux in primary breast cancers, without apparent activation of AMPK, arguing against the involvement of this kinase in mediating the effects of metformin in this clinical context [179]. Similarly, in mouse models of SHH medulloblastoma, it was recently shown that phenformin elicited a potent antitumor effect independently of AMPK and of phosphorylation of the AMPK substrate GLI1 [52,180]. 

Together, all these data suggest that the exact role of AMPK as a mediator of biguanide anticancer action is still unclear and studies using specific loss of function in in vivo models, at different stages of cancer development, are required.

### 4.2. Indirect Antitumor Effects

#### 4.2.1. Effects on Insulin Signaling

The ability of biguanides to lower blood glucose levels through inhibition of hepatic gluconeogenesis and glucose uptake in muscle is thought to contribute to their antitumor properties. Indeed, owing to their glucose-lowering effects, biguanides also reduce circulating levels of insulin and IGF-1. Both hormones bind to receptors that are often expressed at high levels in cancer cells or in cells from which tumors originate, and that activate the oncogenic PAM (Pi3K-AKT-mTOR) pathway, leading to activation of mTOR and promoting cell proliferation and growth [181,182]. Supporting this notion, patients with type II diabetes, who have insulin resistance and thus higher levels of circulating insulin, are at higher risk for various types of cancers due to the mitogenic effects of insulin. Indeed, it has been observed that there is an increased risk of various cancers, including breast [183,184], prostate [185], and colon [186,187] cancers in hyperinsulinemic and obese patients, compared to normal subjects. In this view, the indirect anticancer properties of biguanides are thought to play a role mostly in patients with hyperinsulinemia rather than in subjects that are not insulin resistant at baseline [188].

#### 4.2.2. Effects on the Immune System

According to emerging studies, many of the antitumoral properties of biguanides may rely on their ability to target different components of the immune cells (CD8^+^T cells, Tregs, MDSC, TAM) in the tumor microenvironment.

CD8^+^ T cells: Pearce et al. [189] showed that metformin promotes the generation of CD8^+^ T cells and increases protective immunity against lymphoma in mice, while Ekawa et al. [190] demonstrated that metformin enhances tumor infiltration of CD8^+^T cells, protects them from apoptosis, and promotes the production of IL-2, TNFα, INFγ. Metformin was also shown to increase the effect of anti-PD1-therapy in melanoma cells, by alleviating CD8^+^ T cell suppression through inhibition of cancer cell oxygen consumption and consequent reduction of the hypoxic tumor microenvironment [191]. Additionally, metformin enhances the antitumor immune response of cytotoxic T lymphocytes (CTL) through AMPK-mediated phosphorylation of PD-L1 at S195, which is followed by glycosylation and ERAD-mediated degradation. Therefore, it was shown that the combination of metformin with anti-CTLA4 therapy has a synergistic antitumor effect [192]. Conversely, other studies showed that phenformin decreased INFγ production from CD8^+^ T cells [193] and did not affect tumor infiltration of CTC cells [194].

Thus, given the divergence of these observations, further studies seem to be required to fully understand the effect of biguanides on CD8^+^ T cells.

− Tregs: Biguanides modulate the activity of Tregs, which suppress cytotoxic T cell functions required for tumor elimination. The administration of metformin was shown to decrease the infiltration of Tregs and to reprogram the tumor immune microenvironment in patients with esophageal squamous cell carcinoma [195].
− MDSC: MDSCs are myeloid cell precursors that increase cancer and suppress T and NK cells. Recent works have shown that biguanides inhibit the function of MDSCs in different cancer models and with various mechanisms [196,197,198].
− TAM: Tumor-associated macrophages may contribute to creating an immunosuppressive tumor microenvironment that promotes cancer development. Recent studies have shown that metformin may change the macrophage population toward tumor-suppressive subsets or may inhibit macrophage polarization towards the M2 phenotype in various tumors [199,200].

### 4.3. Variables Affecting the Response to Biguanides in Cancer

The sensitivity of cancer cells to biguanides depends on genetic and microenvironmental factors that allow adaptation to metabolic dysfunctions. Many studies suggest that biguanides alter substrate utilization in the mitochondria [178]. Cancer cells that strongly depend on mitochondrial metabolism and are poorly capable of engaging compensatory glycolysis would be highly sensitive to biguanides. Conversely, leukemia and lymphoma cells markedly depend on the activation of HIF-1a signaling during exposure to biguanides, being resistant to biguanide-induced complex I dysfunction mediated by HIF1α-regulated transcriptional rewiring of glucose metabolism [201]. Cancer cells with mitochondrial defects show a higher sensitivity to biguanides due to the lack of metabolic flexibility at the mitochondrial level. This hypothesis has been confirmed by the evidence of higher phenformin sensitivity in cells harboring complex I mutations [146,153]. Additionally, cancer cells with a defective PGC-1α axis are more sensitive to metformin as well as cells with impaired AMPK signaling [202,203,204], being unable to metabolically adapt to the unfavorable conditions of energy depletion.

The metabolic environment seems also to influence the sensitivity to biguanides. Gui et al. [205] demonstrated that culture media alters the sensitivity of cancer cells to metformin, as cells cultured in DMEM required up to 10 mM metformin to inhibit proliferation, while cells cultured in RPMI media required lower metformin doses. In this scenario, pyruvate was proposed to suppress the anti-proliferative effects of metformin, since cells cultured in DMEM without pyruvate showed increased sensitivity to metformin, while cells cultured in RPMI supplemented with 1 mM pyruvate were less sensitive. Authors proposed that pyruvate modulates complex I dependency by providing an alternative pathway for NAD^+^ regeneration since it acts as an electron acceptor for NAD^+^ regeneration allowing aspartate synthesis [160]. Similarly, glucose availability plays a crucial role in the response to metformin since it was demonstrated that metformin sensitivity in cancer cells was increased upon lowering glucose concentration to 11 mM or upon addition of aspartate (150 μM) in culture media [160]. In another paper from Birsoy and colleagues [146], the authors demonstrated that cancer cells with defects in glucose utilization or complex I function were more sensitive to phenformin. In 0.75 mM glucose media, cell lines with complex I mutations or impaired glucose utilization were 5- to 20-fold more sensitive to phenformin compared to control cancer cell lines. This effect of glucose availability on biguanides sensitivity of cancer cells was further confirmed by another paper where medulloblastoma cells were treated with biguanides in media containing 5.5 mM glucose, corresponding to the average physiological plasma fasting concentration, or 0.75 mM glucose, corresponding to the cancer tissue glucose concentration [52], The authors show that phenformin induced a significant inhibition of cell growth, with a stronger effect at 0.75 mM glucose. While in high glucose conditions the antiproliferative effects of metformin are mediated by the AMPK/LKB1 axis, at low glucose concentrations in the absence of AMPK/LKB1 cells are more sensitive to growth inhibition by metformin, because they are not able to sustain the high energy demand. Dietary limitation through intermittent fasting has been shown to enhance the response to biguanides, and metformin seems to impair tumor growth only when administered during fasting-induced hypoglycemia [205].

Biodistribution and tissue specificity seem also to determine the degree of biguanides accumulation and thus influence their molecular and therapeutical actions. Indeed, the glucose-lowering effect of metformin resulting from inhibition of hepatic gluconeogenesis correlates with the high tissue concentrations that the drug reaches in the liver. Metformin is usually administered orally in diabetic patients, reaching concentrations between 40 and 70 μM in the portal vein, and it accumulates to a larger extent in the gut and liver. This is due to the systemic circulation and to the high level of expression of OCT transporters in these tissues. However, this is not representative of other tissues or organs, where metformin reaches lower micromolar concentrations.

### 4.4. Clinical Studies

Alteration of cellular metabolism is a hallmark of tumor cells, also believed to represent an attractive target for cancer therapy. The best known metabolic alteration in cancer is represented by the so-called Warburg effect, consisting of the transformation of glucose to lactate, regardless of the presence of extracellular oxygen [206]. In more recent years it has been understood that mitochondria are also essential for tumor growth, mostly because of their biosynthetic role rather than their pro-energetic features [207]. In this view, the ability of metformin to inhibit mitochondrial function seems to play an important role in mediating its anti-cancer effect. However, the low availability of metformin in humans at therapeutic antidiabetic doses has pointed to the need to find strategies aimed to maximize its activity and enhance its toxicity toward cancer cells. In this regard, several groups have improved mitochondrial targeting of metformin to achieve therapeutically effective plasma concentrations in cancer patients by modifying its chemical structure, which resulted in mitochondria-targeted metformin analogs with significantly enhanced anti-tumor potential [208,209].

Clinical studies have been performed in diabetic patients where metformin was shown to reduce the incidence of liver, colorectal, breast, and pancreatic cancers and to increase the survival of colorectal, lung, and prostate cancer patients (Table 2). A meta-analysis of ovarian cancer showed a lower incidence and significantly increased survival in patients with diabetes [210]. Another meta-analysis in diabetic patients estimated the relationship between lung cancer incidence and metformin usage and showed a lower risk of cancer in metformin users if compared to non-users [211]. 

More recently, many clinical trials have been developed to investigate the anti-tumoral potential of metformin in nondiabetic patients. Two perspective trials on metformin combinatorial therapy with platinum-based chemotherapy in advanced NSCLC (Non-Small Cell Lung Cancer) showed a composed median overall survival of 17.5 months for patients with KRAS mutations with good tolerability, validating metformin clinical efficacy as adjuvant therapy in this setting [65]. One phase I trial of metformin combinatorial treatment with standard therapy in relapsed refractory acute lymphoblastic leukemia showed an overall response rate (complete and partial responses) of 43% [212]. One randomized, phase II clinical trial of metformin in combination with standard chemotherapy in HER2-negative metastatic breast cancer showed no benefit. Another randomized trial combining metformin with neo-adjuvant chemotherapy in HER2-positive breast cancers (NCT03238495) is still underway. One meta-analysis in pancreatic cancer patients evidenced a significant increase in overall survival in patients at stage I–II and at stage I–IV treated with adjuvant metformin, suggesting a potentially available option for the treatment [213]. However, a randomized phase II study of metformin combinatorial treatment with standard systemic therapy in metastatic pancreatic cancer patients did not show any significant improvement in the clinical outcome [214]. 

Phenformin is currently in phase I clinical trials for combinatorial treatment with dabrafenib and trametinib in patients with BRAFV600E/K-mutated melanoma (NCT03026517).

These studies in normal subjects will unveil the potential of biguanides in oncology, revealing their ability to counteract tumor growth and progression and clarifying the contribution of their systemic effects in the successful clinical outcome that has been observed in diabetic patients treated with this class of drugs.

## 5. Conclusions

Although metformin is prescribed to more than 120 million patients worldwide and almost 3000 papers on biguanides are published every year, how these drugs exert their therapeutic effects is an open question that still begs conclusive answers.

Based on the topics discussed in this article, some conclusions that will find a broad consensus may be drawn and should be taken as general guidelines in future investigations.

1. While inhibition of complex I activity at millimolar concentrations of biguanides is a reproducible phenomenon in vitro and in cell culture, it remains to be fully clarified if this occurs in animal models or in patients taking standard doses of the drugs and, even in such case, if the degree of inhibition is sufficient to mediate a significant biological response when the drugs are given orally at the therapeutic conditions. Except for some tissues, such as the gut and liver, biguanides have been only found at low micromolar concentrations in the body of people taking therapeutic doses of the drugs. Data obtained with overexpression of the budding yeast NDI1, which is often used to formally demonstrate complex I-dependence, may actually be due to effects on other mitochondrial regulators of NAD^+^/NADH ratio and have to be carefully controlled.
2. Activation of AMPK and phosphorylation of its downstream targets are additional well-established events, often believed to be responsible for the therapeutic response to biguanides. As for complex I inhibition, AMPK phosphorylation is generally detected in most cell culture experiments when millimolar doses of biguanides are used. In addition, some data obtained in animal models have shown a certain degree of phosphorylation of AMPK and its targets in response to low levels of biguanides. However, it remains to be fully elucidated if the magnitude of activation reached under therapeutic conditions is biologically meaningful and whether targeted deletion of AMPK truly impairs the response to biguanides in vivo.
3. Any concentration of biguanides, including those that fall within the therapeutic range, causes redox imbalance, with an increased NADH/NAD^+^ ratio. It is still unclear if this is the consequence of the interaction of biguanides with complex I and/or mGPD and/or complex IV and/or other mechanisms. Regardless of the target involved, it should be carefully evaluated to what extent and how redox alterations affect gluconeogenesis or cancer growth. Approaches directed to the selective targeting of the redox state, possibly without causing energy stress, would be needed to properly address this issue.
4. The anticancer effect of biguanides is dependent on several local variables in the tumor microenvironment: drug concentration, nutrient concentration (glucose, pyruvate, amino acids, etc.), and genetic mutations affecting metabolic processes (e.g., respiration, glucose utilization). These aspects need to be fully characterized and evaluated when treating any cells in vivo and in vitro.
5. Biguanides are typically taken orally, and this implies that their effect could be mediated, at least in part, by the interaction with the cells of the GI tract and the commensal microbiota, which may both release molecules involved in an indirect response to the drug. To date, it is still unclear and debated the exact contribution of the gut to the therapeutic properties of biguanides. This issue should also be considered when administering the drug to animal models, by evaluating the effect after parenteral (i.e., i.p., i.v.) administration.

## Figures and Tables

**Table 2:** Clinical trials of biguanides in cancer.

No.	NCT-ID	Title	Status	Treatment	Phase
1	NCT01941953	Metformin and 5-fluorouracil for Refractory Colorectal Cancer	Completed	Metformin Fluorouracil	Phase 2
2	NCT02614339	Effect of Adjunctive Metformin on Recurrence of Non-DM Colorectal Cancer Stage II High-risk/III Colorectal Cancer	Recruiting	Metformin	Phase 3
3	NCT01312467	Trial of Metformin for Colorectal Cancer Risk Reduction for History of Colorectal Adenomas and Elevated BMI	Completed	Metformin HCl	Phase 2
4	NCT01926769	A Phase II Study to Determine the Safety and Efficacy of Second-line Treatment with Metformin and Chemotherapy (FOLFOX6 or FOFIRI) in the Second-Line Treatment of Advanced Colorectal Cancer	Terminated	Metformin	Phase 2
5	NCT01523639	A Randomized, Placebo-controlled, Double-blind Phase II Study Evaluating if Glucophage Can Avoid Liver Injury Due to Chemotherapy Associated Steatosis	Terminated	Metformin	Phase 2
6	NCT01816659	An Open-Labeled Pilot Study of Biomarker Response Following Short-Term Exposure to Metformin	Terminated	Metformin ER	Phase 1
7	NCT03800602	Nivolumab and Metformin in Patients with Treatment Refractory MSS Colorectal Cancer	Recruiting	Metformin Nivolumab	Phase 2
8	NCT01930864	Metformin Plus Irinotecan for Refractory Colorectal Cancer	Recruiting	Metformin Irinotecan	Phase 2
9	NCT03047837	A Randomized, 2 × 2 Factorial Design Biomarker Prevention Trial of Low-dose Aspirin and Metformin in Stage I-III Colorectal Cancer Patients	Recruiting	Aspirin Metformin	Phase 2
10	NCT01440127	Impact of Pretreatment with Metformin on Colorectal Cancer Stem Cells (CCSC) and Related Pharmacodynamic Markers	Terminated	Metformin	Phase 1
11	NCT01340300	Exercise and Metformin in Colorectal and Breast Cancer Survivors	Completed	Metformin, Exercise training, Educational information	Phase 2
12	NCT04033107	High Dose Vitamin C Combined with Metformin in the Treatment of Malignant Tumors	Recruiting	Vitamin C Metformin	Phase 2
13	NCT01632020	Effect of Metformin on Biomarkers of Colorectal Tumor Cell Growth	Terminated	Metformin	Phase 2
14	NCT03359681	Metformin Treatment for Colon Cancer	Recruiting	Metformin	Phase 2
15	NCT02431676	Survivorship Promotion in Reducing IGF-1 Trial	Completed	Metformin, Coach Directed Behavioral Weight Loss, Self-control weight loss	Phase 2
16	NCT02201381	Study of the Safety, Tolerability, and Efficacy of Metabolic Combination Treatments on Cancer	Recruiting	Metformin Atorvastatin Doxycycline Mebendazole	Phase 3
17	NCT02437656	Combination of Metformin with Neoadjuvant Radiochemotherapy in the Treatment of Locally Advanced (METCAP).	Completed	Metformin	Phase 2
18	NCT03053544	Metformin with Neoadjuvant Chemoradiation to Improve Pathologic Responses in Rectal Cancer	Completed	Metformin	Phase 2
19	NCT02473094	Neoadjuvant Metformin in Association with Chemoradiotherapy for Locally Advanced Rectal Cancer	Terminated	Metformin Capecitabine	Phase 2
20	NCT01620593	Castration Compared to Castration Plus Metformin as First-Line Treatment for Patients with Advanced Prostate Cancer	Completed	Metformin	Phase 2
21	NCT02581137	Metformin Hydrochloride in Preventing Oral Cancer in Patients with an Oral Premalignant Lesion	Active	Metformin	Phase 2
22	NCT01447927	Metformin Hydrochloride in Preventing Esophageal Cancer in Patients with Barrett Esophagus	Completed	Metformin	Phase 2
23	NCT03238495	Randomized Trial of Neo-adjuvant Chemotherapy With or Without Metformin for HER2 Positive Operable Breast Cancer (HERMET)	Recruiting	Taxotere, Carboplatin, Herceptin + Pertuzumab Metformin	Phase 2
24	NCT03026517	Clinical Trial of Phenformin in Combination With BRAF Inhibitor + MEK Inhibitor for Patients With BRAF-mutated	Recruiting	Dabrafenib Trametinib Phenformin	Phase 1

## Data Availability

The data presented in this study are available on request from the corresponding author.

## References

[1] Bailey C.J. (2017). Metformin: Historical Overview. Diabetologia.

[2] Currie C.J., Poole C.D., Jenkins-Jones S., Gale E.A.M., Johnson J.A., Morgan C.L. (2012). Mortality after Incident Cancer in People with and without Type 2 Diabetes: Impact of Metformin on Survival. Diabetes Care.

[3] Bowker S.L., Majumdar S.R., Veugelers P., Johnson J.A. (2006). Increased Cancer-Related Mortality for Patients with Type 2 Diabetes Who Use Sulfonylureas or Insulin. Diabetes Care.

[4] Decensi A., Puntoni M., Goodwin P., Cazzaniga M., Gennari A., Bonanni B., Gandini S. (2010). Metformin and Cancer Risk in Diabetic Patients: A Systematic Review and Meta-Analysis. Cancer Prev. Res..

[5] Walker R.S., Linton A.L. (1959). Phenethyldiguanide: A Dangerous Side-Effect. Br. Med. J..

[6] Luft D., Schmülling R.M., Eggstein M. (1978). Lactic Acidosis in Biguanide-Treated Diabetics: A Review of 330 Cases. Diabetologia.

[7] Werner E.A., Bell J. (1922). CCXIV—The Preparation of Methylguanidine, and of Ββ-Dimethylguanidine by the Interaction of Dicyanodiamide, and Methylammonium and Dimethylammonium Chlorides Respectively. J. Chem. Soc. Trans..

[8] Slotta K.H., Tschesche R. (1929). Über Biguanide, II.: Die Blutzucker-Senkende Wirkung Der Biguanide. Ber. Dtsch. Chem. Ges. (A and B Series).

[9] Sterne J. (1957). Du nouveau dans les antidiabetiques. La NN dimethylamine guanyl guanide (NNDG). Maroc. Med..

[10] Graham G.G., Punt J., Arora M., Day R.O., Doogue M.P., Duong J., Furlong T.J., Greenfield J.R., Greenup L.C., Kirkpatrick C.M. (2011). Clinical Pharmacokinetics of Metformin. Clin. Pharm..

[11] Jonker J.W., Wagenaar E., Mol C.A., Buitelaar M., Koepsell H., Smit J.W., Schinkel A.H. (2001). Reduced Hepatic Uptake and Intestinal Excretion of Organic Cations in Mice with a Targeted Disruption of the Organic Cation Transporter 1 (Oct1 [Slc22a1]) Gene. Mol. Cell Biol..

[12] Shu Y., Leabman M.K., Feng B., Mangravite L.M., Huang C.C., Stryke D., Kawamoto M., Johns S.J., DeYoung J., Carlson E. (2003). Evolutionary Conservation Predicts Function of Variants of the Human Organic Cation Transporter, OCT1. Proc. Natl. Acad. Sci. USA.

[13] Nakamichi N., Shima H., Asano S., Ishimoto T., Sugiura T., Matsubara K., Kusuhara H., Sugiyama Y., Sai Y., Miyamoto K.-I. (2013). Involvement of Carnitine/Organic Cation Transporter OCTN1/SLC22A4 in Gastrointestinal Absorption of Metformin. J. Pharm. Sci..

[14] Zhou M., Xia L., Wang J. (2007). Metformin Transport by a Newly Cloned Proton-Stimulated Organic Cation Transporter (Plasma Membrane Monoamine Transporter) Expressed in Human Intestine. Drug Metab. Dispos..

[15] Chen E.C., Liang X., Yee S.W., Geier E.G., Stocker S.L., Chen L., Giacomini K.M. (2015). Targeted Disruption of Organic Cation Transporter 3 Attenuates the Pharmacologic Response to Metformin. Mol. Pharmacol..

[16] Masuda S., Terada T., Yonezawa A., Tanihara Y., Kishimoto K., Katsura T., Ogawa O., Inui K. (2006). Identification and Functional Characterization of a New Human Kidney-Specific H+/Organic Cation Antiporter, Kidney-Specific Multidrug and Toxin Extrusion 2. J. Am. Soc. Nephrol..

[17] Xia L., Engel K., Zhou M., Wang J. (2007). Membrane Localization and PH-Dependent Transport of a Newly Cloned Organic Cation Transporter (PMAT) in Kidney Cells. Am. J. Physiol. Renal. Physiol..

[18] Choi M.-K., Song I.-S. (2008). Organic Cation Transporters and Their Pharmacokinetic and Pharmacodynamic Consequences. Drug Metab. Pharmacokinet.

[19] Hilgendorf C., Ahlin G., Seithel A., Artursson P., Ungell A.-L., Karlsson J. (2007). Expression of Thirty-Six Drug Transporter Genes in Human Intestine, Liver, Kidney, and Organotypic Cell Lines. Drug Metab. Dispos..

[20] Müller J., Lips K.S., Metzner L., Neubert R.H.H., Koepsell H., Brandsch M. (2005). Drug Specificity and Intestinal Membrane Localization of Human Organic Cation Transporters (OCT). Biochem. Pharmacol..

[21] Gong L., Goswami S., Giacomini K.M., Altman R.B., Klein T.E. (2012). Metformin Pathways: Pharmacokinetics and Pharmacodynamics. Pharm. Genom..

[22] Chandel N.S., Avizonis D., Reczek C.R., Weinberg S.E., Menz S., Neuhaus R., Christian S., Haegebarth A., Algire C., Pollak M. (2016). Are Metformin Doses Used in Murine Cancer Models Clinically Relevant?. Cell Metab..

[23] Ma T., Tian X., Zhang B., Li M., Wang Y., Yang C., Wu J., Wei X., Qu Q., Yu Y. (2022). Low-Dose Metformin Targets the Lysosomal AMPK Pathway through PEN2. Nature.

[24] Moonira T., Chachra S.S., Ford B.E., Marin S., Alshawi A., Adam-Primus N.S., Arden C., Al-Oanzi Z.H., Foretz M., Viollet B. (2020). Metformin Lowers Glucose 6-Phosphate in Hepatocytes by Activation of Glycolysis Downstream of Glucose Phosphorylation. J. Biol. Chem..

[25] Tucker G.T., Casey C., Phillips P.J., Connor H., Ward J.D., Woods H.F. (1981). Metformin Kinetics in Healthy Subjects and in Patients with Diabetes Mellitus. Br. J. Clin. Pharmacol..

[26] Lalau J.-D., Lacroix C. (2003). Measurement of Metformin Concentration in Erythrocytes: Clinical Implications. Diabetes Obes. Metab..

[27] Madiraju A.K., Qiu Y., Perry R.J., Rahimi Y., Zhang X.-M., Zhang D., Camporez J.-P.G., Cline G.W., Butrico G.M., Kemp B.E. (2018). Metformin Inhibits Gluconeogenesis via a Redox-Dependent Mechanism In Vivo. Nat. Med..

[28] Bailey C.J., Wilcock C., Scarpello J.H.B. (2008). Metformin and the Intestine. Diabetologia.

[29] Pentikäinen P.J., Neuvonen P.J., Penttilä A. (1979). Pharmacokinetics of Metformin after Intravenous and Oral Administration to Man. Eur. J. Clin. Pharm..

[30] LaMoia T.E., Shulman G.I. (2021). Cellular and Molecular Mechanisms of Metformin Action. Endocr. Rev..

[31] Dowling R.J.O., Lam S., Bassi C., Mouaaz S., Aman A., Kiyota T., Al-Awar R., Goodwin P.J., Stambolic V. (2016). Metformin Pharmacokinetics in Mouse Tumors: Implications for Human Therapy. Cell Metab..

[32] Wilcock C., Bailey C.J. (1994). Accumulation of Metformin by Tissues of the Normal and Diabetic Mouse. Xenobiotica.

[33] Marchetti P., Giannarelli R., di Carlo A., Navalesi R. (1991). Pharmacokinetic Optimisation of Oral Hypoglycaemic Therapy. Clin. Pharm..

[34] Schwartz S., Fonseca V., Berner B., Cramer M., Chiang Y.-K., Lewin A. (2006). Efficacy, Tolerability, and Safety of a Novel Once-Daily Extended-Release Metformin in Patients with Type 2 Diabetes. Diabetes Care.

[35] Ohta K., Inoue K., Yasujima T., Ishimaru M., Yuasa H. (2009). Functional Characteristics of Two Human MATE Transporters: Kinetics of Cimetidine Transport and Profiles of Inhibition by Various Compounds. J. Pharm. Pharm. Sci..

[36] Davidson M.B., Peters A.L. (1997). An Overview of Metformin in the Treatment of Type 2 Diabetes Mellitus. Am. J. Med..

[37] Tanihara Y., Masuda S., Sato T., Katsura T., Ogawa O., Inui K.-I. (2007). Substrate Specificity of MATE1 and MATE2-K, Human Multidrug and Toxin Extrusions/H^+^-Organic Cation Antiporters. Biochem. Pharmacol..

[38] Shapiro S.L., Parrino V.A., Freedman L. (1959). Hypoglycemic Agents. I.^1^ Chemical Properties of β-Phenethylbiguanide.^2^ A New Hypoglycemic Agent^3^. J. Am. Chem. Soc..

[39] Appleyard M.V.C.L., Murray K.E., Coates P.J., Wullschleger S., Bray S.E., Kernohan N.M., Fleming S., Alessi D.R., Thompson A.M. (2012). Phenformin as Prophylaxis and Therapy in Breast Cancer Xenografts. Br. J. Cancer.

[40] Daugan M., Dufaÿ Wojcicki A., d’Hayer B., Boudy V. (2016). Metformin: An Anti-Diabetic Drug to Fight Cancer. Pharmacol. Res..

[41] Hawley S.A., Ross F.A., Chevtzoff C., Green K.A., Evans A., Fogarty S., Towler M.C., Brown L.J., Ogunbayo O.A., Evans A.M. (2010). Use of Cells Expressing Gamma Subunit Variants to Identify Diverse Mechanisms of AMPK Activation. Cell Metab..

[42] Shitara Y., Nakamichi N., Norioka M., Shima H., Kato Y., Horie T. (2013). Role of Organic Cation/Carnitine Transporter 1 in Uptake of Phenformin and Inhibitory Effect on Complex I Respiration in Mitochondria. Toxicol. Sci..

[43] Bridges H.R., Sirviö V.A., Agip A.-N.A., Hirst J. (2016). Molecular Features of Biguanides Required for Targeting of Mitochondrial Respiratory Complex I and Activation of AMP-Kinase. BMC Biol..

[44] Sogame Y., Kitamura A., Yabuki M., Komuro S., Takano M. (2013). Transport of Biguanides by Human Organic Cation Transporter OCT2. Biomed. Pharm..

[45] Beckmann R. (1968). The Fate of Biguanides in Man. Ann. N. Y. Acad. Sci..

[46] Shah R.R., Evans D.A., Oates N.S., Idle J.R., Smith R.L. (1985). The Genetic Control of Phenformin 4-Hydroxylation. J. Med. Genet.

[47] Alkalay D., Khemani L., Wagner W.E., Bartlett M.F. (1975). Pharmacokinetics of Phenformin in Man. J. Clin. Pharmacol..

[48] Matin S.B., Karam J.H., Forsham P.H., Knight J.B. (1974). Determination of Phenformin in Biological Fluids Using Chemical Ionization Mass Spectrometry. Biomed. Mass Spectrom..

[49] Nattrass M., Sizer K., Alberti K.G. (1980). Correlation of Plasma Phenformin Concentration with Metabolic Effects in Normal Subjects. Clin. Sci..

[50] Marchetti P., Navalesi R. (1989). Pharmacokinetic-Pharmacodynamic Relationships of Oral Hypoglycaemic Agents. An Update. Clin. Pharm..

[51] Karam J.H., Matin S.B., Forsham P.H. (1975). Antidiabetic Drugs after the University Group Diabetes Program (UGDP). Annu. Rev. Pharmacol..

[52] Di Magno L., Manni S., Di Pastena F., Coni S., Macone A., Cairoli S., Sambucci M., Infante P., Moretti M., Petroni M. (2020). Phenformin Inhibits Hedgehog-Dependent Tumor Growth through a Complex I-Independent Redox/Corepressor Module. Cell Rep..

[53] Huang X., Wullschleger S., Shpiro N., McGuire V.A., Sakamoto K., Woods Y.L., McBurnie W., Fleming S., Alessi D.R. (2008). Important Role of the LKB1-AMPK Pathway in Suppressing Tumorigenesis in PTEN-Deficient Mice. Biochem. J..

[54] Shackelford D.B., Abt E., Gerken L., Vasquez D.S., Seki A., Leblanc M., Wei L., Fishbein M.C., Czernin J., Mischel P.S. (2013). LKB1 Inactivation Dictates Therapeutic Response of Non-Small Cell Lung Cancer to the Metabolism Drug Phenformin. Cancer Cell.

[55] Bando K., Ochiai S., Kunimatsu T., Deguchi J., Kimura J., Funabashi H., Seki T. (2010). Comparison of Potential Risks of Lactic Acidosis Induction by Biguanides in Rats. Regul. Toxicol. Pharmacol..

[56] Wick A.N., Bolinger R., Shapiro S., Clarke D.W., Ungar G., Kruger F.A., Volk B.W. (1960). Laboratory Studies with Phenformin: Panel Discussion. Diabetes.

[57] Sogame Y., Kitamura A., Yabuki M., Komuro S. (2011). Liver Uptake of Biguanides in Rats. Biomed. Pharm..

[58] Conlay L.A., Karam J.H., Matin S.B., Loewenstein J.E. (1977). Serum Phenformin Concentrations in Patients with Phenformin-Associated Lactic Acidosis. Diabetes.

[59] Bosisio E., Kienle M.G., Galli G., Ciconali M., Negri A., Sessa A., Morosati S., Sirtori C.R. (1981). Defective Hydroxylation of Phenformin as a Determinant of Drug Toxicity. Diabetes.

[60] Ungar G., Freedman L., Shapiro S.L. (1957). Pharmacological Studies of a New Oral Hypoglycemic Drug. Proc. Soc. Exp. Biol. Med..

[61] Zhu Z., Jiang W., Thompson M.D., Echeverria D., McGinley J.N., Thompson H.J. (2015). Effects of Metformin, Buformin, and Phenformin on the Post-Initiation Stage of Chemically Induced Mammary Carcinogenesis in the Rat. Cancer Prev. Res..

[62] Beckmann R., Lintz W., Schmidt-Böthelt E. (1971). Evaluation of a Sustained Release Form of the Oral Antidiabetic Butylbiguanide (Silubin Retard). Eur. J. Clin. Pharmacol..

[63] Beckmann R. (1966). The Mechanism of Action of the Biguanides. Ger. Med. Mon..

[64] Beckmann R., Hübner G. (1965). On the pharmacokinetics of 1-butyl-biguanide hydrochloride and the prolonged-action form of this substance. Arzneimittelforschung.

[65] Garrett E.R., Tsau J., Hinderling P.H. (1972). Application of Ion-Pair Methods to Drug Extraction from Biological Fluids. II. Quantitative Determination of Biguanides in Biological Fluids and Comparison of Protein Binding Estimates. J. Pharm. Sci..

[66] Lintz W., Berger W., Aenishaenslin W., Kutova V., Baerlocher C., Kapp J.P., Beckmann R. (1974). Butylbiguanide Concentration in Plasma, Liver, and Intestine after Intravenous and Oral Administration to Man. Eur. J. Clin. Pharmacol..

[67] Yoh Y.J. (1967). Distribution of N-Butylbiguanide-^14^C Hydrochloride in Mouse Tissues. Jpn. J. Pharmacol..

[68] Haller H., Strauzenberg S.E. (1966). A contribution to the method of determination of biguanides, creatinine and creatine in the urine. Arztl. Forsch.

[69] Losert W., Kolb K.H., Bitterling G. (1972). Distribution of 1-butyl-biguanide- 14 C in rats and guinea pigs. Arzneimittelforschung.

[70] Caspary W.F., Creutzfeldt W. (1973). Inhibition of Intestinal Amino Acid Transport by Blood Sugar Lowering Biguanides. Diabetologia.

[71] Khan M.A.B., Hashim M.J., King J.K., Govender R.D., Mustafa H., Al Kaabi J. (2020). Epidemiology of Type 2 Diabetes—Global Burden of Disease and Forecasted Trends. J. Epidemiol. Glob. Health.

[72] Magnusson I., Rothman D.L., Katz L.D., Shulman R.G., Shulman G.I. (1992). Increased Rate of Gluconeogenesis in Type II Diabetes Mellitus. A 13C Nuclear Magnetic Resonance Study. J. Clin. Investig..

[73] DeFronzo R.A., Ferrannini E., Groop L., Henry R.R., Herman W.H., Holst J.J., Hu F.B., Kahn C.R., Raz I., Shulman G.I. (2015). Type 2 Diabetes Mellitus. Nat. Rev. Dis. Primers.

[74] He L., Wondisford F.E. (2015). Metformin Action: Concentrations Matter. Cell Metab..

[75] Nasri H., Rafieian-Kopaei M. (2014). Metformin: Current Knowledge. J. Res. Med. Sci..

[76] Eisenreich A., Leppert U. (2017). Update on the Protective Renal Effects of Metformin in Diabetic Nephropathy. Curr. Med. Chem..

[77] Lord J.M., Flight I.H.K., Norman R.J. (2003). Metformin in Polycystic Ovary Syndrome: Systematic Review and Meta-Analysis. BMJ.

[78] Selvin E., Bolen S., Yeh H.-C., Wiley C., Wilson L.M., Marinopoulos S.S., Feldman L., Vassy J., Wilson R., Bass E.B. (2008). Cardiovascular Outcomes in Trials of Oral Diabetes Medications: A Systematic Review. Arch. Intern. Med..

[79] Salvatore T., Galiero R., Caturano A., Vetrano E., Rinaldi L., Coviello F., Di Martino A., Albanese G., Marfella R., Sardu C. (2021). Effects of Metformin in Heart Failure: From Pathophysiological Rationale to Clinical Evidence. Biomolecules.

[80] Hundal R.S., Krssak M., Dufour S., Laurent D., Lebon V., Chandramouli V., Inzucchi S.E., Schumann W.C., Petersen K.F., Landau B.R. (2000). Mechanism by Which Metformin Reduces Glucose Production in Type 2 Diabetes. Diabetes.

[81] El-Mir M.Y., Nogueira V., Fontaine E., Avéret N., Rigoulet M., Leverve X. (2000). Dimethylbiguanide Inhibits Cell Respiration via an Indirect Effect Targeted on the Respiratory Chain Complex I. J. Biol. Chem..

[82] Owen M.R., Doran E., Halestrap A.P. (2000). Evidence That Metformin Exerts Its Anti-Diabetic Effects through Inhibition of Complex 1 of the Mitochondrial Respiratory Chain. Biochem. J..

[83] Hirst J. (2013). Mitochondrial Complex I. Annu. Rev. Biochem..

[84] Bridges H.R., Jones A.J.Y., Pollak M.N., Hirst J. (2014). Effects of Metformin and Other Biguanides on Oxidative Phosphorylation in Mitochondria. Biochem. J..

[85] Stephenne X., Foretz M., Taleux N., van der Zon G.C., Sokal E., Hue L., Viollet B., Guigas B. (2011). Metformin Activates AMP-Activated Protein Kinase in Primary Human Hepatocytes by Decreasing Cellular Energy Status. Diabetologia.

[86] Zhou G., Myers R., Li Y., Chen Y., Shen X., Fenyk-Melody J., Wu M., Ventre J., Doebber T., Fujii N. (2001). Role of AMP-Activated Protein Kinase in Mechanism of Metformin Action. J. Clin. Investig..

[87] Shaw R.J., Lamia K.A., Vasquez D., Koo S.-H., Bardeesy N., Depinho R.A., Montminy M., Cantley L.C. (2005). The Kinase LKB1 Mediates Glucose Homeostasis in Liver and Therapeutic Effects of Metformin. Science.

[88] Foretz M., Hébrard S., Leclerc J., Zarrinpashneh E., Soty M., Mithieux G., Sakamoto K., Andreelli F., Viollet B. (2010). Metformin Inhibits Hepatic Gluconeogenesis in Mice Independently of the LKB1/AMPK Pathway via a Decrease in Hepatic Energy State. J. Clin. Investig..

[89] Hunter R.W., Hughey C.C., Lantier L., Sundelin E.I., Peggie M., Zeqiraj E., Sicheri F., Jessen N., Wasserman D.H., Sakamoto K. (2018). Metformin Reduces Liver Glucose Production by Inhibition of Fructose-1-6-Bisphosphatase. Nat. Med..

[90] Miller R.A., Chu Q., Xie J., Foretz M., Viollet B., Birnbaum M.J. (2013). Biguanides Suppress Hepatic Glucagon Signalling by Decreasing Production of Cyclic AMP. Nature.

[91] Hardie D.G. (2013). Metformin-Acting through Cyclic AMP as Well as AMP?. Cell Metab..

[92] Hasenour C.M., Ridley D.E., Hughey C.C., James F.D., Donahue E.P., Shearer J., Viollet B., Foretz M., Wasserman D.H. (2014). 5-Aminoimidazole-4-Carboxamide-1-β-D-Ribofuranoside (AICAR) Effect on Glucose Production, but Not Energy Metabolism, Is Independent of Hepatic AMPK In Vivo. J. Biol. Chem..

[93] Cokorinos E.C., Delmore J., Reyes A.R., Albuquerque B., Kjøbsted R., Jørgensen N.O., Tran J.-L., Jatkar A., Cialdea K., Esquejo R.M. (2017). Activation of Skeletal Muscle AMPK Promotes Glucose Disposal and Glucose Lowering in Non-Human Primates and Mice. Cell Metab..

[94] Vial G., Detaille D., Guigas B. (2019). Role of Mitochondria in the Mechanism(s) of Action of Metformin. Front. Endocrinol..

[95] Fontaine E. (2018). Metformin-Induced Mitochondrial Complex I Inhibition: Facts, Uncertainties, and Consequences. Front. Endocrinol..

[96] Carvalho C., Correia S., Santos M.S., Seiça R., Oliveira C.R., Moreira P.I. (2008). Metformin Promotes Isolated Rat Liver Mitochondria Impairment. Mol. Cell Biochem..

[97] Kelley D.E., He J., Menshikova E.V., Ritov V.B. (2002). Dysfunction of Mitochondria in Human Skeletal Muscle in Type 2 Diabetes. Diabetes.

[98] Morino K., Petersen K.F., Dufour S., Befroy D., Frattini J., Shatzkes N., Neschen S., White M.F., Bilz S., Sono S. (2005). Reduced Mitochondrial Density and Increased IRS-1 Serine Phosphorylation in Muscle of Insulin-Resistant Offspring of Type 2 Diabetic Parents. J. Clin. Investig..

[99] Petersen K.F., Dufour S., Befroy D., Garcia R., Shulman G.I. (2004). Impaired Mitochondrial Activity in the Insulin-Resistant Offspring of Patients with Type 2 Diabetes. N. Engl. J. Med..

[100] Ritov V.B., Menshikova E.V., He J., Ferrell R.E., Goodpaster B.H., Kelley D.E. (2005). Deficiency of Subsarcolemmal Mitochondria in Obesity and Type 2 Diabetes. Diabetes.

[101] Liesa M., Shirihai O.S. (2013). Mitochondrial Dynamics in the Regulation of Nutrient Utilization and Energy Expenditure. Cell Metab..

[102] Youle R.J., van der Bliek A.M. (2012). Mitochondrial Fission, Fusion, and Stress. Science.

[103] Twig G., Elorza A., Molina A.J.A., Mohamed H., Wikstrom J.D., Walzer G., Stiles L., Haigh S.E., Katz S., Las G. (2008). Fission and Selective Fusion Govern Mitochondrial Segregation and Elimination by Autophagy. EMBO J..

[104] Yamada T., Murata D., Adachi Y., Itoh K., Kameoka S., Igarashi A., Kato T., Araki Y., Huganir R.L., Dawson T.M. (2018). Mitochondrial Stasis Reveals P62-Mediated Ubiquitination in Parkin-Independent Mitophagy and Mitigates Nonalcoholic Fatty Liver Disease. Cell Metab..

[105] Wang Y., An H., Liu T., Qin C., Sesaki H., Guo S., Radovick S., Hussain M., Maheshwari A., Wondisford F.E. (2019). Metformin Improves Mitochondrial Respiratory Activity through Activation of AMPK. Cell Rep..

[106] Larsen S., Rabøl R., Hansen C.N., Madsbad S., Helge J.W., Dela F. (2012). Metformin-Treated Patients with Type 2 Diabetes Have Normal Mitochondrial Complex I Respiration. Diabetologia.

[107] Madiraju A.K., Erion D.M., Rahimi Y., Zhang X.-M., Braddock D.T., Albright R.A., Prigaro B.J., Wood J.L., Bhanot S., MacDonald M.J. (2014). Metformin Suppresses Gluconeogenesis by Inhibiting Mitochondrial Glycerophosphate Dehydrogenase. Nature.

[108] Mráček T., Drahota Z., Houštěk J. (2013). The Function and the Role of the Mitochondrial Glycerol-3-Phosphate Dehydrogenase in Mammalian Tissues. Biochim. Biophys. Acta.

[109] Baur J.A., Birnbaum M.J. (2014). Control of Gluconeogenesis by Metformin: Does Redox Trump Energy Charge?. Cell Metab..

[110] Saheki T., Iijima M., Li M.X., Kobayashi K., Horiuchi M., Ushikai M., Okumura F., Meng X.J., Inoue I., Tajima A. (2007). Citrin/Mitochondrial Glycerol-3-Phosphate Dehydrogenase Double Knock-out Mice Recapitulate Features of Human Citrin Deficiency. J. Biol. Chem..

[111] Alshawi A., Agius L. (2019). Low Metformin Causes a More Oxidized Mitochondrial NADH/NAD Redox State in Hepatocytes and Inhibits Gluconeogenesis by a Redox-Independent Mechanism. J. Biol. Chem..

[112] Calza G., Nyberg E., Mäkinen M., Soliymani R., Cascone A., Lindholm D., Barborini E., Baumann M., Lalowski M., Eriksson O. (2018). Lactate-Induced Glucose Output Is Unchanged by Metformin at a Therapeutic Concentration—A Mass Spectrometry Imaging Study of the Perfused Rat Liver. Front. Pharmacol..

[113] MacDonald M.J., Ansari I.-U.H., Longacre M.J., Stoker S.W. (2021). Metformin’s Therapeutic Efficacy in the Treatment of Diabetes Does Not Involve Inhibition of Mitochondrial Glycerol Phosphate Dehydrogenase. Diabetes.

[114] LaMoia T.E., Butrico G.M., Kalpage H.A., Goedeke L., Hubbard B.T., Vatner D.F., Gaspar R.C., Zhang X.-M., Cline G.W., Nakahara K. (2022). Metformin, Phenformin, and Galegine Inhibit Complex IV Activity and Reduce Glycerol-Derived Gluconeogenesis. Proc. Natl. Acad. Sci. USA.

[115] Xie D., Chen F., Zhang Y., Shi B., Song J., Chaudhari K., Yang S.-H., Zhang G.J., Sun X., Taylor H.S. (2022). Let-7 Underlies Metformin-Induced Inhibition of Hepatic Glucose Production. Proc. Natl. Acad. Sci. USA.

[116] Zhang C.-S., Hawley S.A., Zong Y., Li M., Wang Z., Gray A., Ma T., Cui J., Feng J.-W., Zhu M. (2017). Fructose-1,6-Bisphosphate and Aldolase Mediate Glucose Sensing by AMPK. Nature.

[117] Sum C.F., Webster J.M., Johnson A.B., Catalano C., Cooper B.G., Taylor R. (1992). The Effect of Intravenous Metformin on Glucose Metabolism during Hyperglycaemia in Type 2 Diabetes. Diabet Med..

[118] Bonora E., Cigolini M., Bosello O., Zancanaro C., Capretti L., Zavaroni I., Coscelli C., Butturini U. (1984). Lack of Effect of Intravenous Metformin on Plasma Concentrations of Glucose, Insulin, C-Peptide, Glucagon and Growth Hormone in Non-Diabetic Subjects. Curr. Med. Res. Opin..

[119] Oh J., Chung H., Park S.-I., Yi S.J., Jang K., Kim A.H., Yoon J., Cho J.-Y., Yoon S.H., Jang I.-J. (2016). Inhibition of the Multidrug and Toxin Extrusion (MATE) Transporter by Pyrimethamine Increases the Plasma Concentration of Metformin but Does Not Increase Antihyperglycaemic Activity in Humans. Diabetes Obes. Metab..

[120] Buse J.B., DeFronzo R.A., Rosenstock J., Kim T., Burns C., Skare S., Baron A., Fineman M. (2016). The Primary Glucose-Lowering Effect of Metformin Resides in the Gut, Not the Circulation: Results From Short-Term Pharmacokinetic and 12-Week Dose-Ranging Studies. Diabetes Care.

[121] Wu H., Esteve E., Tremaroli V., Khan M.T., Caesar R., Mannerås-Holm L., Ståhlman M., Olsson L.M., Serino M., Planas-Fèlix M. (2017). Metformin Alters the Gut Microbiome of Individuals with Treatment-Naive Type 2 Diabetes, Contributing to the Therapeutic Effects of the Drug. Nat. Med..

[122] Koffert J.P., Mikkola K., Virtanen K.A., Andersson A.-M.D., Faxius L., Hällsten K., Heglind M., Guiducci L., Pham T., Silvola J.M.U. (2017). Metformin Treatment Significantly Enhances Intestinal Glucose Uptake in Patients with Type 2 Diabetes: Results from a Randomized Clinical Trial. Diabetes Res. Clin. Pract..

[123] Bahler L., Holleman F., Chan M.-W., Booij J., Hoekstra J.B., Verberne H.J. (2017). 18F-FDG Uptake in the Colon Is Modulated by Metformin but Not Associated with Core Body Temperature and Energy Expenditure. PLoS ONE.

[124] Andersen A., Lund A., Knop F.K., Vilsbøll T. (2018). Glucagon-like Peptide 1 in Health and Disease. Nat. Rev. Endocrinol..

[125] Maida A., Lamont B.J., Cao X., Drucker D.J. (2011). Metformin Regulates the Incretin Receptor Axis via a Pathway Dependent on Peroxisome Proliferator-Activated Receptor-α in Mice. Diabetologia.

[126] Preiss D., Dawed A., Welsh P., Heggie A., Jones A.G., Dekker J., Koivula R., Hansen T.H., Stewart C., Holman R.R. (2017). Sustained Influence of Metformin Therapy on Circulating Glucagon-like Peptide-1 Levels in Individuals with and without Type 2 Diabetes. Diabetes Obes. Metab..

[127] Bahne E., Sun E.W.L., Young R.L., Hansen M., Sonne D.P., Hansen J.S., Rohde U., Liou A.P., Jackson M.L., de Fontgalland D. (2018). Metformin-Induced Glucagon-like Peptide-1 Secretion Contributes to the Actions of Metformin in Type 2 Diabetes. JCI Insight.

[128] Migoya E.M., Bergeron R., Miller J.L., Snyder R.N.K., Tanen M., Hilliard D., Weiss B., Larson P., Gutierrez M., Jiang G. (2010). Dipeptidyl Peptidase-4 Inhibitors Administered in Combination with Metformin Result in an Additive Increase in the Plasma Concentration of Active GLP-1. Clin. Pharmacol. Ther..

[129] Wu T., Thazhath S.S., Bound M.J., Jones K.L., Horowitz M., Rayner C.K. (2014). Mechanism of Increase in Plasma Intact GLP-1 by Metformin in Type 2 Diabetes: Stimulation of GLP-1 Secretion or Reduction in Plasma DPP-4 Activity?. Diabetes Res. Clin. Pract..

[130] Golay A. (2008). Metformin and Body Weight. Int. J. Obes..

[131] Lee A., Morley J.E. (1998). Metformin Decreases Food Consumption and Induces Weight Loss in Subjects with Obesity with Type II Non-Insulin-Dependent Diabetes. Obes. Res..

[132] Coll A.P., Chen M., Taskar P., Rimmington D., Patel S., Tadross J.A., Cimino I., Yang M., Welsh P., Virtue S. (2020). GDF15 Mediates the Effects of Metformin on Body Weight and Energy Balance. Nature.

[133] Day E.A., Ford R.J., Smith B.K., Mohammadi-Shemirani P., Morrow M.R., Gutgesell R.M., Lu R., Raphenya A.R., Kabiri M., McArthur A.G. (2019). Metformin-Induced Increases in GDF15 Are Important for Suppressing Appetite and Promoting Weight Loss. Nat. Metab..

[134] Baek S.J., Eling T. (2019). Growth Differentiation Factor 15 (GDF15): A Survival Protein with Therapeutic Potential in Metabolic Diseases. Pharmacol. Ther..

[135] Baek S.J., Okazaki R., Lee S.-H., Martinez J., Kim J.-S., Yamaguchi K., Mishina Y., Martin D.W., Shoieb A., McEntee M.F. (2006). Nonsteroidal Anti-Inflammatory Drug-Activated Gene-1 over Expression in Transgenic Mice Suppresses Intestinal Neoplasia. Gastroenterology.

[136] Chrysovergis K., Wang X., Kosak J., Lee S.-H., Kim J.S., Foley J.F., Travlos G., Singh S., Baek S.J., Eling T.E. (2014). NAG-1/GDF-15 Prevents Obesity by Increasing Thermogenesis, Lipolysis and Oxidative Metabolism. Int. J. Obes..

[137] Sun L., Xie C., Wang G., Wu Y., Wu Q., Wang X., Liu J., Deng Y., Xia J., Chen B. (2018). Gut Microbiota and Intestinal FXR Mediate the Clinical Benefits of Metformin. Nat. Med..

[138] Forslund K., Hildebrand F., Nielsen T., Falony G., Le Chatelier E., Sunagawa S., Prifti E., Vieira-Silva S., Gudmundsdottir V., Pedersen H.K. (2015). Disentangling Type 2 Diabetes and Metformin Treatment Signatures in the Human Gut Microbiota. Nature.

[139] Nosadini R., Avogaro A., Trevisan R., Valerio A., Tessari P., Duner E., Tiengo A., Velussi M., Del Prato S., De Kreutzenberg S. (1987). Effect of Metformin on Insulin-Stimulated Glucose Turnover and Insulin Binding to Receptors in Type II Diabetes. Diabetes Care.

[140] Galuska D., Nolte L.A., Zierath J.R., Wallberg-Henriksson H. (1994). Effect of Metformin on Insulin-Stimulated Glucose Transport in Isolated Skeletal Muscle Obtained from Patients with NIDDM. Diabetologia.

[141] Kjøbsted R., Munk-Hansen N., Birk J.B., Foretz M., Viollet B., Björnholm M., Zierath J.R., Treebak J.T., Wojtaszewski J.F.P. (2017). Enhanced Muscle Insulin Sensitivity after Contraction/Exercise Is Mediated by AMPK. Diabetes.

[142] Kjøbsted R., Kristensen J.M., Birk J.B., Eskesen N.O., Kido K., Andersen N.R., Larsen J.K., Foretz M., Viollet B., Nielsen F. (2022). Metformin Improves Glycemia Independently of Skeletal Muscle AMPK via Enhanced Intestinal Glucose Clearance. bioRxiv.

[143] Yu J.G., Kruszynska Y.T., Mulford M.I., Olefsky J.M. (1999). A Comparison of Troglitazone and Metformin on Insulin Requirements in Euglycemic Intensively Insulin-Treated Type 2 Diabetic Patients. Diabetes.

[144] Evans J.M.M., Donnelly L.A., Emslie-Smith A.M., Alessi D.R., Morris A.D. (2005). Metformin and Reduced Risk of Cancer in Diabetic Patients. BMJ.

[145] Wheaton W.W., Weinberg S.E., Hamanaka R.B., Soberanes S., Sullivan L.B., Anso E., Glasauer A., Dufour E., Mutlu G.M., Budigner G.S. (2014). Metformin Inhibits Mitochondrial Complex I of Cancer Cells to Reduce Tumorigenesis. eLife.

[146] Birsoy K., Possemato R., Lorbeer F.K., Bayraktar E.C., Thiru P., Yucel B., Wang T., Chen W.W., Clish C.B., Sabatini D.M. (2014). Metabolic Determinants of Cancer Cell Sensitivity to Glucose Limitation and Biguanides. Nature.

[147] Igelmann S., Lessard F., Uchenunu O., Bouchard J., Fernandez-Ruiz A., Rowell M.-C., Lopes-Paciencia S., Papadopoli D., Fouillen A., Ponce K.J. (2021). A Hydride Transfer Complex Reprograms NAD Metabolism and Bypasses Senescence. Mol. Cell.

[148] Schöckel L., Glasauer A., Basit F., Bitschar K., Truong H., Erdmann G., Algire C., Hägebarth A., Willems P.H., Kopitz C. (2015). Targeting Mitochondrial Complex I Using BAY 87-2243 Reduces Melanoma Tumor Growth. Cancer Metab.

[149] Zhang C.-S., Jiang B., Li M., Zhu M., Peng Y., Zhang Y.-L., Wu Y.-Q., Li T.Y., Liang Y., Lu Z. (2014). The Lysosomal V-ATPase-Ragulator Complex Is a Common Activator for AMPK and MTORC1, Acting as a Switch between Catabolism and Anabolism. Cell Metab..

[150] Guigas B., Detaille D., Chauvin C., Batandier C., De Oliveira F., Fontaine E., Leverve X. (2004). Metformin Inhibits Mitochondrial Permeability Transition and Cell Death: A Pharmacological In Vitro Study. Biochem. J..

[151] Sancho P., Burgos-Ramos E., Tavera A., Bou Kheir T., Jagust P., Schoenhals M., Barneda D., Sellers K., Campos-Olivas R., Graña O. (2015). MYC/PGC-1α Balance Determines the Metabolic Phenotype and Plasticity of Pancreatic Cancer Stem Cells. Cell Metab..

[152] Cheng G., Zielonka J., Ouari O., Lopez M., McAllister D., Boyle K., Barrios C.S., Weber J.J., Johnson B.D., Hardy M. (2016). Mitochondria-Targeted Analogues of Metformin Exhibit Enhanced Antiproliferative and Radiosensitizing Effects in Pancreatic Cancer Cells. Cancer Res..

[153] Momcilovic M., Jones A., Bailey S.T., Waldmann C.M., Li R., Lee J.T., Abdelhady G., Gomez A., Holloway T., Schmid E. (2019). In Vivo Imaging of Mitochondrial Membrane Potential in Non-Small-Cell Lung Cancer. Nature.

[154] Orr A.L., Ashok D., Sarantos M.R., Ng R., Shi T., Gerencser A.A., Hughes R.E., Brand M.D. (2014). Novel Inhibitors of Mitochondrial Sn-Glycerol 3-Phosphate Dehydrogenase. PLoS ONE.

[155] Christensen M.M.H., Brasch-Andersen C., Green H., Nielsen F., Damkier P., Beck-Nielsen H., Brosen K. (2011). The Pharmacogenetics of Metformin and Its Impact on Plasma Metformin Steady-State Levels and Glycosylated Hemoglobin A1c. Pharm. Genom..

[156] Scheen A.J. (1996). Clinical Pharmacokinetics of Metformin. Clin. Pharm..

[157] Thakur S., Daley B., Gaskins K., Vasko V.V., Boufraqech M., Patel D., Sourbier C., Reece J., Cheng S.-Y., Kebebew E. (2018). Metformin Targets Mitochondrial Glycerophosphate Dehydrogenase to Control Rate of Oxidative Phosphorylation and Growth of Thyroid Cancer In Vitro and In Vivo. Clin. Cancer Res..

[158] Xie J., Ye J., Cai Z., Luo Y., Zhu X., Deng Y., Feng Y., Liang Y., Liu R., Han Z. (2020). GPD1 Enhances the Anticancer Effects of Metformin by Synergistically Increasing Total Cellular Glycerol-3-Phosphate. Cancer Res..

[159] Liu S., Fu S., Wang G., Cao Y., Li L., Li X., Yang J., Li N., Shan Y., Cao Y. (2021). Glycerol-3-Phosphate Biosynthesis Regenerates Cytosolic NAD^+^ to Alleviate Mitochondrial Disease. Cell Metab..

[160] Gui D.Y., Sullivan L.B., Luengo A., Hosios A.M., Bush L.N., Gitego N., Davidson S.M., Freinkman E., Thomas C.J., Vander Heiden M.G. (2016). Environment Dictates Dependence on Mitochondrial Complex I for NAD^+^ and Aspartate Production and Determines Cancer Cell Sensitivity to Metformin. Cell Metab..

[161] Vara-Ciruelos D., Dandapani M., Hardie D.G. (2020). AMP-Activated Protein Kinase: Friend or Foe in Cancer?. Annu. Rev. Cancer Biol..

[162] Eichner L.J., Brun S.N., Herzig S., Young N.P., Curtis S.D., Shackelford D.B., Shokhirev M.N., Leblanc M., Vera L.I., Hutchins A. (2019). Genetic Analysis Reveals AMPK Is Required to Support Tumor Growth in Murine Kras-Dependent Lung Cancer Models. Cell Metab..

[163] Zhang C.-S., Li M., Ma T., Zong Y., Cui J., Feng J.-W., Wu Y.-Q., Lin S.-Y., Lin S.-C. (2016). Metformin Activates AMPK through the Lysosomal Pathway. Cell Metab..

[164] Zou Z., Tao T., Li H., Zhu X. (2020). MTOR Signaling Pathway and MTOR Inhibitors in Cancer: Progress and Challenges. Cell Biosci..

[165] Sabatini D.M. (2006). MTOR and Cancer: Insights into a Complex Relationship. Nat. Rev. Cancer.

[166] Um S.H., D’Alessio D., Thomas G. (2006). Nutrient Overload, Insulin Resistance, and Ribosomal Protein S6 Kinase 1, S6K1. Cell Metab..

[167] Wullschleger S., Loewith R., Hall M.N. (2006). TOR Signaling in Growth and Metabolism. Cell.

[168] Dowling R.J.O., Zakikhani M., Fantus I.G., Pollak M., Sonenberg N. (2007). Metformin Inhibits Mammalian Target of Rapamycin-Dependent Translation Initiation in Breast Cancer Cells. Cancer Res..

[169] Gwinn D.M., Shackelford D.B., Egan D.F., Mihaylova M.M., Mery A., Vasquez D.S., Turk B.E., Shaw R.J. (2008). AMPK Phosphorylation of Raptor Mediates a Metabolic Checkpoint. Mol. Cell.

[170] Kalender A., Selvaraj A., Kim S.Y., Gulati P., Brûlé S., Viollet B., Kemp B.E., Bardeesy N., Dennis P., Schlager J.J. (2010). Metformin, Independent of AMPK, Inhibits MTORC1 in a Rag GTPase-Dependent Manner. Cell Metab..

[171] Jones R.G., Plas D.R., Kubek S., Buzzai M., Mu J., Xu Y., Birnbaum M.J., Thompson C.B. (2005). AMP-Activated Protein Kinase Induces a P53-Dependent Metabolic Checkpoint. Mol. Cell.

[172] Buzzai M., Jones R.G., Amaravadi R.K., Lum J.J., DeBerardinis R.J., Zhao F., Viollet B., Thompson C.B. (2007). Systemic Treatment with the Antidiabetic Drug Metformin Selectively Impairs P53-Deficient Tumor Cell Growth. Cancer Res..

[173] Faubert B., Boily G., Izreig S., Griss T., Samborska B., Dong Z., Dupuy F., Chambers C., Fuerth B.J., Viollet B. (2013). AMPK Is a Negative Regulator of the Warburg Effect and Suppresses Tumor Growth In Vivo. Cell Metab..

[174] Moiseeva O., Deschênes-Simard X., St-Germain E., Igelmann S., Huot G., Cadar A.E., Bourdeau V., Pollak M.N., Ferbeyre G. (2013). Metformin Inhibits the Senescence-Associated Secretory Phenotype by Interfering with IKK/NF-ΚB Activation. Aging Cell.

[175] Hirsch H.A., Iliopoulos D., Struhl K. (2013). Metformin Inhibits the Inflammatory Response Associated with Cellular Transformation and Cancer Stem Cell Growth. Proc. Natl. Acad. Sci. USA.

[176] Tan X.-L., Bhattacharyya K.K., Dutta S.K., Bamlet W.R., Rabe K.G., Wang E., Smyrk T.C., Oberg A.L., Petersen G.M., Mukhopadhyay D. (2015). Metformin Suppresses Pancreatic Tumor Growth with Inhibition of NFκB/STAT3 Inflammatory Signaling. Pancreas.

[177] Qi X., Xu W., Xie J., Wang Y., Han S., Wei Z., Ni Y., Dong Y., Han W. (2016). Metformin Sensitizes the Response of Oral Squamous Cell Carcinoma to Cisplatin Treatment through Inhibition of NF-ΚB/HIF-1α Signal Axis. Sci. Rep..

[178] Liu X., Romero I.L., Litchfield L.M., Lengyel E., Locasale J.W. (2016). Metformin Targets Central Carbon Metabolism and Reveals Mitochondrial Requirements in Human Cancers. Cell Metab..

[179] Lord S.R., Cheng W.-C., Liu D., Gaude E., Haider S., Metcalf T., Patel N., Teoh E.J., Gleeson F., Bradley K. (2018). Integrated Pharmacodynamic Analysis Identifies Two Metabolic Adaption Pathways to Metformin in Breast Cancer. Cell Metab..

[180] Di Magno L., Basile A., Coni S., Manni S., Sdruscia G., D’Amico D., Antonucci L., Infante P., De Smaele E., Cucchi D. (2016). The Energy Sensor AMPK Regulates Hedgehog Signaling in Human Cells through a Unique Gli1 Metabolic Checkpoint. Oncotarget.

[181] Schultze S.M., Hemmings B.A., Niessen M., Tschopp O. (2012). PI3K/AKT, MAPK and AMPK Signalling: Protein Kinases in Glucose Homeostasis. Expert Rev. Mol. Med..

[182] Latres E., Amini A.R., Amini A.A., Griffiths J., Martin F.J., Wei Y., Lin H.C., Yancopoulos G.D., Glass D.J. (2005). Insulin-like Growth Factor-1 (IGF-1) Inversely Regulates Atrophy-Induced Genes via the Phosphatidylinositol 3-Kinase/Akt/Mammalian Target of Rapamycin (PI3K/Akt/MTOR) Pathway. J. Biol. Chem..

[183] Pritchard K.I., Shepherd L.E., Chapman J.-A.W., Norris B.D., Cantin J., Goss P.E., Dent S.F., Walde D., Vandenberg T.A., Findlay B. (2011). Randomized Trial of Tamoxifen versus Combined Tamoxifen and Octreotide LAR Therapy in the Adjuvant Treatment of Early-Stage Breast Cancer in Postmenopausal Women: NCIC CTG MA.14. J. Clin. Oncol..

[184] Goodwin P.J., Ennis M., Pritchard K.I., Trudeau M.E., Koo J., Taylor S.K., Hood N. (2012). Insulin- and Obesity-Related Variables in Early-Stage Breast Cancer: Correlations and Time Course of Prognostic Associations. J. Clin. Oncol..

[185] Di Sebastiano K.M., Pinthus J.H., Duivenvoorden W.C.M., Mourtzakis M. (2018). Glucose Impairments and Insulin Resistance in Prostate Cancer: The Role of Obesity, Nutrition and Exercise. Obes. Rev..

[186] Ma J., Giovannucci E., Pollak M., Leavitt A., Tao Y., Gaziano J.M., Stampfer M.J. (2004). A Prospective Study of Plasma C-Peptide and Colorectal Cancer Risk in Men. J. Natl. Cancer Inst..

[187] Wolpin B.M., Meyerhardt J.A., Chan A.T., Ng K., Chan J.A., Wu K., Pollak M.N., Giovannucci E.L., Fuchs C.S. (2009). Insulin, the Insulin-like Growth Factor Axis, and Mortality in Patients with Nonmetastatic Colorectal Cancer. J. Clin. Oncol..

[188] Campagnoli C., Pasanisi P., Abbà C., Ambroggio S., Biglia N., Brucato T., Colombero R., Danese S., Donadio M., Venturelli E. (2012). Effect of Different Doses of Metformin on Serum Testosterone and Insulin in Non-Diabetic Women with Breast Cancer: A Randomized Study. Clin. Breast Cancer.

[189] Pearce E.L., Walsh M.C., Cejas P.J., Harms G.M., Shen H., Wang L.-S., Jones R.G., Choi Y. (2009). Enhancing CD8 T-Cell Memory by Modulating Fatty Acid Metabolism. Nature.

[190] Eikawa S., Nishida M., Mizukami S., Yamazaki C., Nakayama E., Udono H. (2015). Immune-Mediated Antitumor Effect by Type 2 Diabetes Drug, Metformin. Proc. Natl. Acad. Sci. USA.

[191] Scharping N.E., Menk A.V., Whetstone R.D., Zeng X., Delgoffe G.M. (2017). Efficacy of PD-1 Blockade Is Potentiated by Metformin-Induced Reduction of Tumor Hypoxia. Cancer Immunol. Res..

[192] Cha J.-H., Yang W.-H., Xia W., Wei Y., Chan L.-C., Lim S.-O., Li C.-W., Kim T., Chang S.-S., Lee H.-H. (2018). Metformin Promotes Antitumor Immunity via Endoplasmic-Reticulum-Associated Degradation of PD-L1. Mol. Cell.

[193] Blagih J., Coulombe F., Vincent E.E., Dupuy F., Galicia-Vázquez G., Yurchenko E., Raissi T.C., van der Windt G.J.W., Viollet B., Pearce E.L. (2015). The Energy Sensor AMPK Regulates T Cell Metabolic Adaptation and Effector Responses In Vivo. Immunity.

[194] Kim S.H., Li M., Trousil S., Zhang Y., Pasca di Magliano M., Swanson K.D., Zheng B. (2017). Phenformin Inhibits Myeloid-Derived Suppressor Cells and Enhances the Anti-Tumor Activity of PD-1 Blockade in Melanoma. J. Investig. Dermatol..

[195] Wang S., Lin Y., Xiong X., Wang L., Guo Y., Chen Y., Chen S., Wang G., Lin P., Chen H. (2020). Low-Dose Metformin Reprograms the Tumor Immune Microenvironment in Human Esophageal Cancer: Results of a Phase II Clinical Trial. Clin. Cancer Res..

[196] Baumann T., Dunkel A., Schmid C., Schmitt S., Hiltensperger M., Lohr K., Laketa V., Donakonda S., Ahting U., Lorenz-Depiereux B. (2020). Regulatory Myeloid Cells Paralyze T Cells through Cell-Cell Transfer of the Metabolite Methylglyoxal. Nat. Immunol..

[197] Uehara T., Eikawa S., Nishida M., Kunisada Y., Yoshida A., Fujiwara T., Kunisada T., Ozaki T., Udono H. (2019). Metformin Induces CD11b^+^-Cell-Mediated Growth Inhibition of an Osteosarcoma: Implications for Metabolic Reprogramming of Myeloid Cells and Anti-Tumor Effects. Int. Immunol..

[198] Xu P., Yin K., Tang X., Tian J., Zhang Y., Ma J., Xu H., Xu Q., Wang S. (2019). Metformin Inhibits the Function of Granulocytic Myeloid-Derived Suppressor Cells in Tumor-Bearing Mice. Biomed. Pharm..

[199] Ma Q., Gu J.-T., Wang B., Feng J., Yang L., Kang X.-W., Duan P., Sun X., Liu P.-J., Wang J.-C. (2019). PlGF Signaling and Macrophage Repolarization Contribute to the Anti-Neoplastic Effect of Metformin. Eur. J. Pharmacol..

[200] Sloot Y.J.E., Rabold K., Netea M.G., Smit J.W.A., Hoogerbrugge N., Netea-Maier R.T. (2019). Effect of PTEN Inactivating Germline Mutations on Innate Immune Cell Function and Thyroid Cancer-Induced Macrophages in Patients with PTEN Hamartoma Tumor Syndrome. Oncogene.

[201] Khan H., Anshu A., Prasad A., Roy S., Jeffery J., Kittipongdaja W., Yang D.T., Schieke S.M. (2019). Metabolic Rewiring in Response to Biguanides Is Mediated by MROS/HIF-1a in Malignant Lymphocytes. Cell Rep..

[202] Deblois G., St-Pierre J., Giguère V. (2013). The PGC-1/ERR Signaling Axis in Cancer. Oncogene.

[203] Audet-Walsh É., Papadopoli D.J., Gravel S.-P., Yee T., Bridon G., Caron M., Bourque G., Giguère V., St-Pierre J. (2016). The PGC-1α/ERRα Axis Represses One-Carbon Metabolism and Promotes Sensitivity to Anti-Folate Therapy in Breast Cancer. Cell Rep..

[204] Chaube B., Malvi P., Singh S.V., Mohammad N., Meena A.S., Bhat M.K. (2015). Targeting Metabolic Flexibility by Simultaneously Inhibiting Respiratory Complex I and Lactate Generation Retards Melanoma Progression. Oncotarget.

[205] Elgendy M., Cirò M., Hosseini A., Weiszmann J., Mazzarella L., Ferrari E., Cazzoli R., Curigliano G., DeCensi A., Bonanni B. (2019). Combination of Hypoglycemia and Metformin Impairs Tumor Metabolic Plasticity and Growth by Modulating the PP2A-GSK3β-MCL-1 Axis. Cancer Cell.

[206] Liberti M.V., Locasale J.W. (2016). The Warburg Effect: How Does It Benefit Cancer Cells?. Trends Biochem. Sci..

[207] Missiroli S., Perrone M., Genovese I., Pinton P., Giorgi C. (2020). Cancer Metabolism and Mitochondria: Finding Novel Mechanisms to Fight Tumours. EBioMedicine.

[208] Kalyanaraman B., Cheng G., Hardy M., Ouari O., Sikora A., Zielonka J., Dwinell M. (2017). Mitochondria-Targeted Metformins: Anti-Tumour and Redox Signalling Mechanisms. Interface Focus.

[209] Boyle K.A., Van Wickle J., Hill R.B., Marchese A., Kalyanaraman B., Dwinell M.B. (2018). Mitochondria-Targeted Drugs Stimulate Mitophagy and Abrogate Colon Cancer Cell Proliferation. J. Biol. Chem..

[210] Shih A.J., Menzin A., Whyte J., Lovecchio J., Liew A., Khalili H., Bhuiya T., Gregersen P.K., Lee A.T. (2018). Identification of Grade and Origin Specific Cell Populations in Serous Epithelial Ovarian Cancer by Single Cell RNA-Seq. PLoS ONE.

[211] Yao L., Liu M., Huang Y., Wu K., Huang X., Zhao Y., He W., Zhang R. (2019). Metformin Use and Lung Cancer Risk in Diabetic Patients: A Systematic Review and Meta-Analysis. Dis. Markers.

[212] Trucco M., Barredo J.C., Goldberg J., Leclerc G.M., Hale G.A., Gill J., Setty B., Smith T., Lush R., Lee J.K. (2018). A Phase I Window, Dose Escalating and Safety Trial of Metformin in Combination with Induction Chemotherapy in Relapsed Refractory Acute Lymphoblastic Leukemia: Metformin with Induction Chemotherapy of Vincristine, Dexamethasone, PEG-Asparaginase, and Doxorubicin. Pediatr. Blood Cancer.

[213] Wan G., Sun X., Li F., Wang X., Li C., Li H., Yu X., Cao F. (2018). Survival Benefit of Metformin Adjuvant Treatment for Pancreatic Cancer Patients: A Systematic Review and Meta-Analysis. Cell Physiol. Biochem..

[214] Reni M., Dugnani E., Cereda S., Belli C., Balzano G., Nicoletti R., Liberati D., Pasquale V., Scavini M., Maggiora P. (2016). (Ir)Relevance of Metformin Treatment in Patients with Metastatic Pancreatic Cancer: An Open-Label, Randomized Phase II Trial. Clin. Cancer Res..

